# Immunological and microbial shifts in the aging rhesus macaque lung during nontuberculous mycobacterial infection

**DOI:** 10.1128/mbio.00829-24

**Published:** 2024-05-21

**Authors:** Isaac R. Cinco, Ethan G. Napier, Nicholas S. Rhoades, Michael H. Davies, Derek B. Allison, Steven G. Kohama, Luiz Bermudez, Kevin Winthrop, Cristina Fuss, Eliot R. Spindel, Ilhem Messaoudi

**Affiliations:** 1 Department of Microbiology, Immunology, and Molecular Genetics, College of Medicine, University of Kentucky, Lexington, Kentucky, USA; 2 Division of Neuroscience, Oregon National Primate Research Center, Oregon Health and Science University, Beaverton, Oregon, USA; 3 Department of Pathology and Laboratory Medicine, College of Medicine, University of Kentucky, Lexington, Kentucky, USA; 4 Department of Microbiology, College of Sciences, Oregon State University, Corvallis, Oregon, USA; 5 Division of Infectious Diseases, School of Medicine, Oregon Health and Science University, Portland, Oregon, USA; 6 Division of Infectious Diseases, School of Public Health, Oregon Health and Science University, Portland, Oregon, USA; 7 Department of Radiology and Biomedical Imaging, Yale University School of Medicine, New Haven, Connecticut, USA; University of Michigan-Ann Arbor, Ann Arbor, Michigan, USA

**Keywords:** aging, lung, lung immunology, *M. avium *complex, single-cell RNA sequencing, microbiome, rhesus macaque

## Abstract

Nontuberculous mycobacteria (NTM) are environmentally ubiquitous organisms that predominately cause NTM pulmonary disease (NTMPD) in individuals over the age of 65. The incidence of NTMPD has increased in the U.S., exceeding that of *Mycobacterium tuberculosis*. However, the mechanisms leading to higher susceptibility and severity of NTMPD with aging are poorly defined in part due to the lack of animal models that accurately recapitulate human disease. Here, we compared bacterial load, microbial communities, and host responses longitudinally between three young (two female and one male) and two aged (two female) rhesus macaques inoculated with *Mycobacterium avium* subsp. *hominissuis* (MAH) in the right caudal lobe. Unilateral infection resulted in a low bacterial load in both young and aged animals confined to the infected side. Although a robust inflammatory response was only observed in the inoculated lung, immune cell infiltration and antigen-specific T cells were detected in both lungs. Computed tomography, gross pathology, and histopathology revealed increased disease severity and persistence of bacterial DNA in aged animals. Additional analyses showed the translocation of gut and oral-pharyngeal bacterial DNA into the lower respiratory microbiome. Finally, single-cell RNA sequencing revealed a heightened inflammatory response to MAH infection by alveolar macrophages in aged animals. These data are consistent with the model that increased disease severity in the aged is mediated by a dysregulated macrophage response that may be sustained through persistent antigen presence.

**IMPORTANCE:**

Nontuberculous mycobacteria (NTM) are emerging as pathogens of high consequence, as cases of NTM pulmonary disease (NTMPD) have exceeded those of *Mycobacterium tuberculosis*. NTMPD can be debilitating, particularly in patients over 65 years of age, as it causes chronic cough and fatigue requiring prolonged treatments with antibiotics. The underlying mechanisms of this increased disease severity with age are poorly understood, hampering the development of therapeutics and vaccines. Here, we use a rhesus macaque model to investigate the impact of age on host-NTM interactions. This work shows that aging is associated with increased disease severity and bacterial persistence in aged rhesus macaques, thus providing a preclinical model to develop and test novel therapeutics and interventions.

## INTRODUCTION

Nontuberculous mycobacteria (NTM) pulmonary disease (NTMPD) is caused primarily by *Mycobacterium avium* complex (MAC; composed of *M. avium* and *Mycobacterium intracellulare* among other species), *Mycobacterium abscessus*, and *Mycobacterium kansasii* (1–6). These ubiquitous bacteria reside in soil and water sources and can infect the lung after inhalation of aerosols, aspiration of liquid, or secretion from the upper respiratory tract (7). Despite widespread exposure, NTMPD is predominantly observed in older individuals and in those with underlying lung diseases, such as bronchiectasis of various etiologies, chronic obstructive pulmonary disease, and cystic fibrosis (5, 8–11). This chronic and sometimes debilitating lung disease is characterized by coughing, shortness of breath, exhaustion, weight/appetite loss, and mild fever. The prevalence of NTMPD has increased from 12 to 18 cases per 100,000 persons between 2008 and 2015 (12), now exceeding that of *Mycobacterium tuberculosis* in the U.S. (5). Given the increasing proportion of individuals older than 65 years, it is anticipated that prevalence of NTMPD will continue to rise (13, 14).

Clinical and genome-wide association studies suggest that T-helper 1 (Th1) T-cell responses are necessary for an effective immune response against NTM (15). For instance, peripheral blood mononuclear cells from patients with a history of NTM infection produced lower levels of Th1 cytokines (IFNγ and TNFα) in response to mycobacterial antigens (16). Other studies highlighted critical roles for toll-like receptor (TLR) signaling and innate immunity in the control of NTM infection (17–21). Recent studies have suggested that the dysbiosis of the respiratory microbiome is associated with the acquisition of NTM (22–24). Specifically, a decrease in *Leptotrichia*, *Streptococcus*, and *Veilonella* in the sputum of women with NTM was observed (23), though cause versus effect is unclear. On the other hand, the bronchoalveolar lavage (BAL) microbiome of patients with NTMPD is enriched in *Ralstonia*, *Clavibacter*, and *Enterobacter* (24). However, the role that the BAL microbiome plays in susceptibility to NTM infection remains unknown due to the lack of longitudinal studies that capture pre-infection time points.

Studies carried out using immune-competent rodents have provided important insight into the pathogenesis of disseminated NTM infection (25, 26). A study utilizing an aerosol route of MAC infection in a modified Cornell-like murine model identified IFN as a critical cytokine for the defense against disseminated NTM disease (27). However, disseminated NTM is largely different from NTMPD where infection is limited to the lung (28–30), making it unclear whether these findings are relevant in a pulmonary disease setting. Additionally, the specific-pathogen-free status of rodents results in considerable differences in the immunological and microbial respiratory landscapes (31–35) compared to humans. To date, there is a lack of rodent models that recapitulate human NTMPD or age-mediated exacerbation in disease severity.

To overcome the limitations of clinical and rodent model studies, we utilized a rhesus macaque model of *M. avium* infection (36). Rhesus macaques’ respiratory and immune systems share significant homology with those of humans and recapitulate the hallmarks of human aging (36–38). As described for humans, the lung microbiome of healthy rhesus macaques is predominantly composed of microbes from the phyla Actinobacteria (*Tropherema*), Firmicutes (*Streptococcus* and *Veillonella*), Bacteriodetes (*Prevotella*), Proteobacteria (*Neisseria*), and Fusobacteria (*Fusobacterium*) (39). We previously showed that intrabronchial inoculation of rhesus macaques with *Mycobacterium avium* subsp. *hominissuis* (MAH) resulted in the development of bronchiectasis and the formation of granulomatous lesions in the lung of one of the three infected animals (36). The disease was associated with a dampened Th1 response indicated by lower frequencies of IFNγ + T cells and a Th2 bias evidenced by higher levels of antibodies and IL-6 (36). Herein, we leveraged the rhesus macaque model to investigate the mechanisms underlying increased NTMPD severity with age by profiling immune and microbial changes in young and aged animals prior to and following infection with *M. avium* subsp. *hominissuis*.

## RESULTS

### Aged rhesus macaques exhibit severe NTMPD following MAH infection

**Fig 1:**
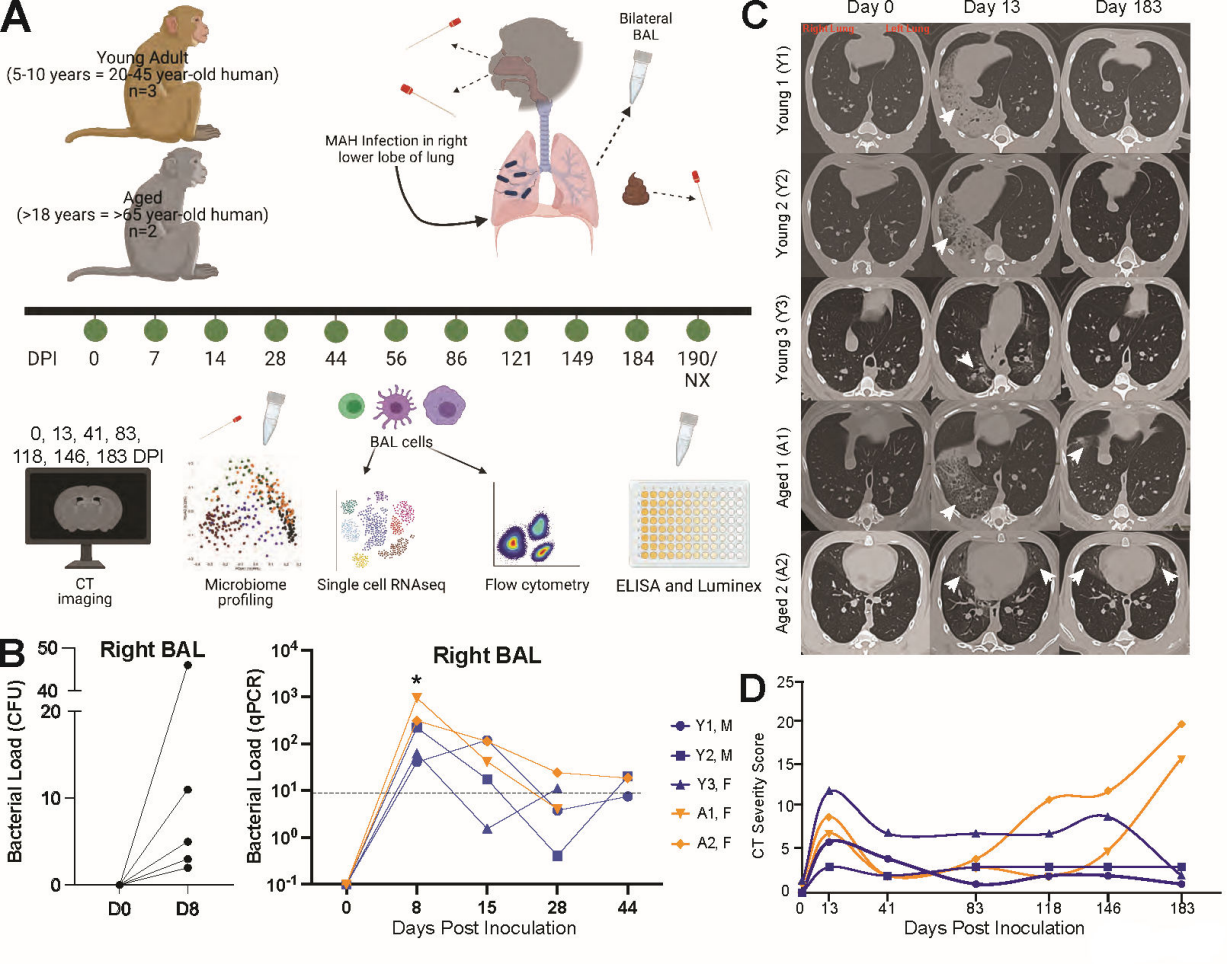
Age-related differences in disease severity are observed in rhesus macaques. (**A**) Experimental design. Three young adult (5–10 years) and two aged (>18 years) rhesus macaques were inoculated in the right caudal lobe with a bolus of 6.8 × 10^8^ CFU of MAH. Nasal, oral, fecal, and BAL samples were collected at the indicated days post-inoculation. BAL cells were probed via microbiome profiling, scRNA-seq, flow cytometry, ELISA, and Luminex assays. Microbiome profiling was done on the nasal, oral, and fecal swabs. Computed tomography (CT) scans were obtained, and severity scores were determined at 0, 13, 41, 83, 118, 145, and 183 DPI. (**B**) Bacterial load was measured in the BAL via CFU after 8 weeks of incubation onto Lowenstein-Jensen agar plates and quantitative real-time PCR specific for the IS1311 sequence in *M. avium*. The dashed line represents the limit of detection. Each time point for all animals was compared to 0 DPI (*P < 0.05). No live MAH or DNA was detected in the left BAL samples. (**C**) Representative CT scans of the young and aged animals before inoculation (0 DPI), at the height of inflammation (13 DPI), and close to necropsy (183 DPI). White arrowheads point out inflammation. (**D**) CT scans were analyzed by a blinded cardiothoracic radiologist and reported as severity scores at the indicated days post-inoculation. Data from young animals (Y1–Y3) are plotted in blue and aged animals (A1 and A2) are plotted in orange. M indicates males and F indicates females.

Rhesus macaques (Table S1) were infected with MAH (Fig. 1A). BAL samples were collected separately from the right and left lungs to measure immune responses, MAH burden, and lung microbiome (Fig. 1A). Bacterial burden was determined by bacterial culture and real-time quantitative PCR (qPCR). Live MAH were detected only in the right (but not left) BAL 8 days post-infection (DPI) measured by colony-forming units (CFU) (Fig. 1B, left panel). Bacterial load in the BAL measured by qPCR peaked at 8 DPI followed by a rapid decline in both young and aged animals to undetectable levels after 44 DPI (Fig. 1B, right panel).

**Fig 2:**
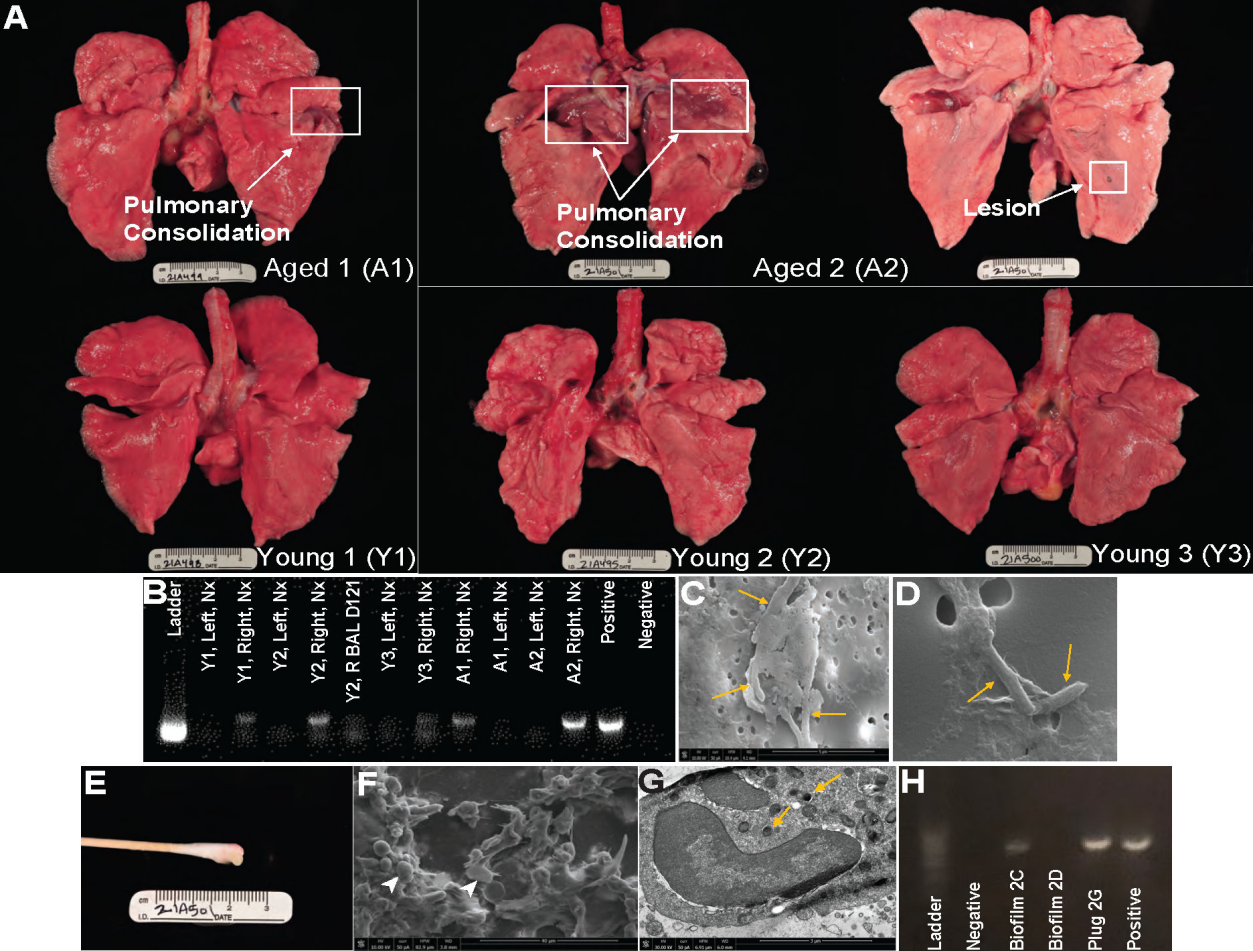
Aged animals have damaged lungs with *M. avium* residing in biofilms and macrophages. (**A**) Gross lung pathology exhibiting pulmonary consolidations and lesions in the aged compared to young animals at necropsy. (**B**) PCR gel of lung swab cultures at necropsy (Nx) specific for MAH 16S rRNA gene (16S R-GGGTCCCCGTCAATTCCTTT; 16S F-CTGGCTCAGGATGAACGCT). (**C**) SEM showing *M. avium* embedded in matrix-rich biofilm in the right lung. (**D**) Scanning electron microscopy showing *M. avium* embedded in left lung biofilm. (**E**) Aggregate of host cells from the right lung of A2. (**F**) SEM of the host cell aggregate showing myeloid cells (white arrowheads). (**G**) Transmission electron microscopy showing a lung macrophage from the aggregate with several viable *M. avium* inside of cytoplasm vacuoles. Yellow arrows show bacteria. (**H**) PCR gel of biofilms shown in panels C, D, and G.

Animals underwent computed tomography (CT) scans to assess radiographic changes in response to infection and to define disease severity. Considerable inflammation was observed in all animals at 13 DPI (Fig. 1C). While radiographic changes improved in both young and aged animals at 41 DPI, inflammatory changes were detected again at 118 DPI in aged animals and remained until 183 DPI (necropsy time point) (Fig. 1C and D). Increased disease severity in the lungs from aged animals was evident at necropsy with signs of pulmonary consolidation and lesions (Fig. 2A). Despite the lack of evidence of MAH by qPCR of the BAL, its presence was confirmed by PCR in swabs obtained from right but not left lungs from only aged animals at necropsy, suggesting the persistence of MAH in biofilm (Fig. 2B). Indeed, scanning electron microscopy (SEM) revealed the presence of biofilms in both the right and left lungs of animal aged 2 (A2) (Fig. 2C and D). Additionally, an aggregate of host cells was collected from the trachea of A2 (Fig. 2E) and found to harbor myeloid cells (Fig. 2F), notably alveolar macrophages (AM) containing MAH (Fig. 2G). DNA extracted from the biofilms obtained from A2 was confirmed to harbor MAH using PCR (Fig. 2H).

### Increased immune infiltrates are detected in the lungs of aged animals with MAH infection

**Fig 3:**
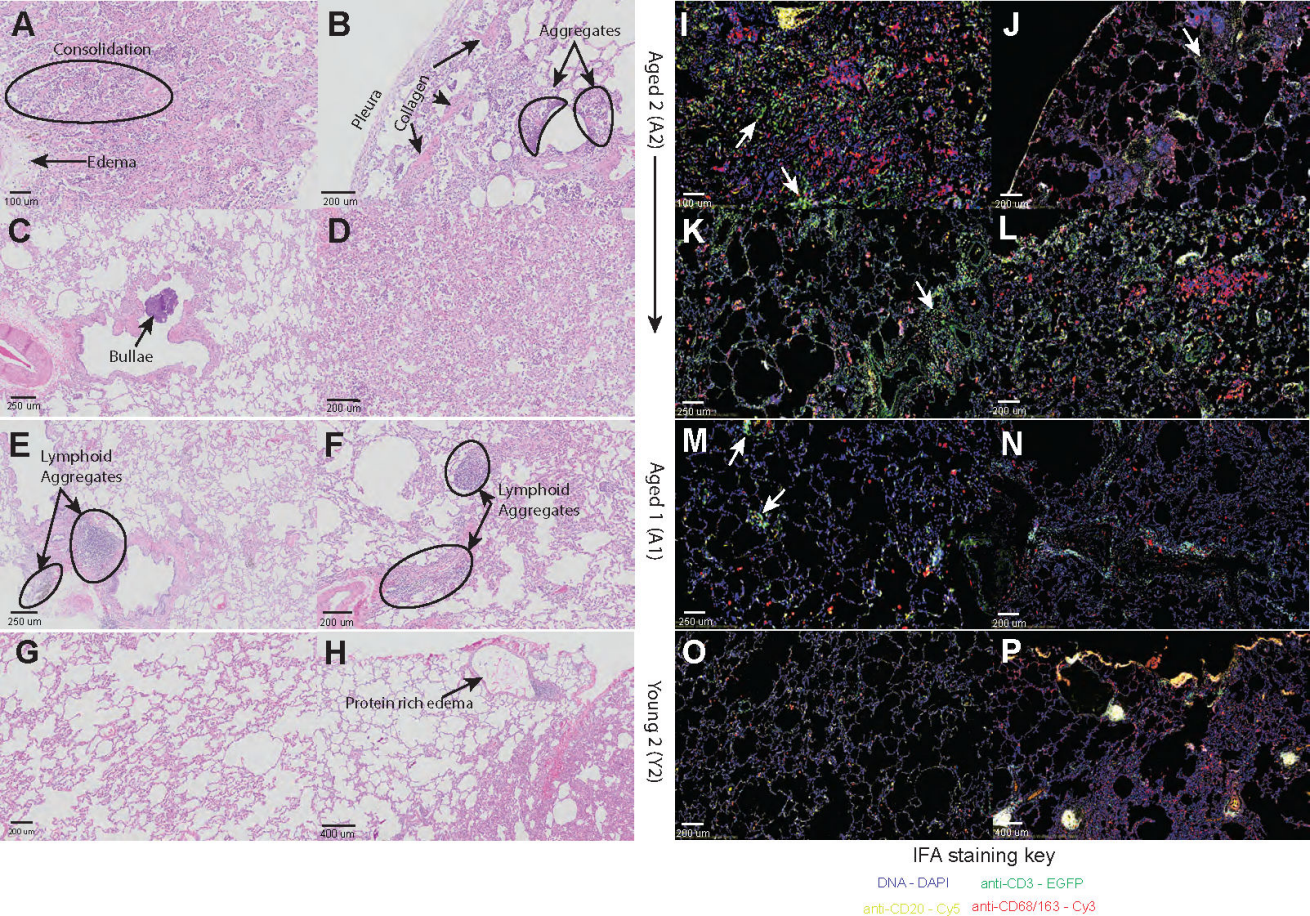
Age-related pulmonary changes and inflammation observed in MAH-infected nonhuman primate (NHP) lung samples. H and E stains at the indicated magnifications of aged 2 (A2) (**A**) right accessory lobe 1, (**B**) right caudal lobe, (**C**) left caudal lobe, and (**D**) right accessory lobe 2; aged 1 (A1) (**E**) right cranial lobe and (**F**) right caudal lobe; young 2 (Y2) (**G**) right middle lobe and (**H**) right caudal lobe. IFA stains at 20× magnification of A2 (**I**) right accessory lobe 1, (**J**) right caudal lobe, (**K**) left caudal lobe, and (**L**) right accessory lobe 2; A1 (**M**) right cranial lobe and (**N**) right caudal lobe; Y2 (**O**) right middle lobe and (**P**) right caudal lobe. The fluorescent channels used are 4′,6-diamidino-2-phenylindole (DAPI) stain for a general DNA stain (blue); enhanced green fluorescent protein (EGFP) conjugated to anti-CD3 for T-cell identification (green); Cyanine 5 (Cy5) conjugated to anti-CD20 for B-cell identification (yellow); and Cyanine 3 (Cy3) conjugated to anti-CD68/163 for the identification of macrophages and monocytes (red).

Hematoxylin and eosin (H and E) staining showed immune infiltrates (lymphocytes, neutrophils, and eosinophils), lymphoid aggregates, and interstitial fibrosis (collagen deposition, pulmonary edema, and bronchopneumonia) in the lungs from aged animals (Fig. 3A through F; Fig. S1A through C). More specifically, pulmonary consolidation, inflammation, interstitial edema, and interstitial fibrosis were observed in the right accessory lobe in animal A2 (Fig. 3A). The left caudal lobe of A2 also harbored a bulla containing neutrophil aggregates (Fig. 3C). A1 exhibited interstitial and peribronchiolar inflammation indicated by multiple lymphoid aggregates (Fig. 3E and F). In contrast, the lungs of young animals exhibited minimal inflammation and little pulmonary edema, with unobstructed airways and pleura (Fig. 3G; Fig. S1D and E), except for the right caudal lobe of Y2, which contained a small region of protein-rich fluid (Fig. 3H).

Immunofluorescence analysis (Fig. 3I through P; Fig. S1F through Q and S2) and enumeration of immune infiltrates were conducted using the HALO Image Analysis platform allowing the identification of T cells expressing cell surface CD3 staining (Fig. S1G and S2A); B cells with characteristic cell surface CD20 clustering (Fig. S1H and S2B); and macrophages with CD68/CD163 cytoplasmic and membranous localization (Fig. S1I and S2C through F). This analysis revealed a statistically significant increase in the frequency of macrophages and T cells in lung sections from aged compared to young animals (Fig. S1R). Infiltrating T cells were found in the right accessory, right caudal, and left caudal sections of A2 and in the right cranial and caudal lobes of A1 (Fig. 3I through N; Fig. S1K through M). Sections from young animals showed healthy lung tissue (Fig. 3O; Fig. S1N and O), except for Y2, which had some lymphoid aggregates near the region of protein-rich fluid in the right caudal lobe (Fig. 3P).

### MAH infection results in significant inflammation in the BAL from young and aged animals

**Fig 4:**
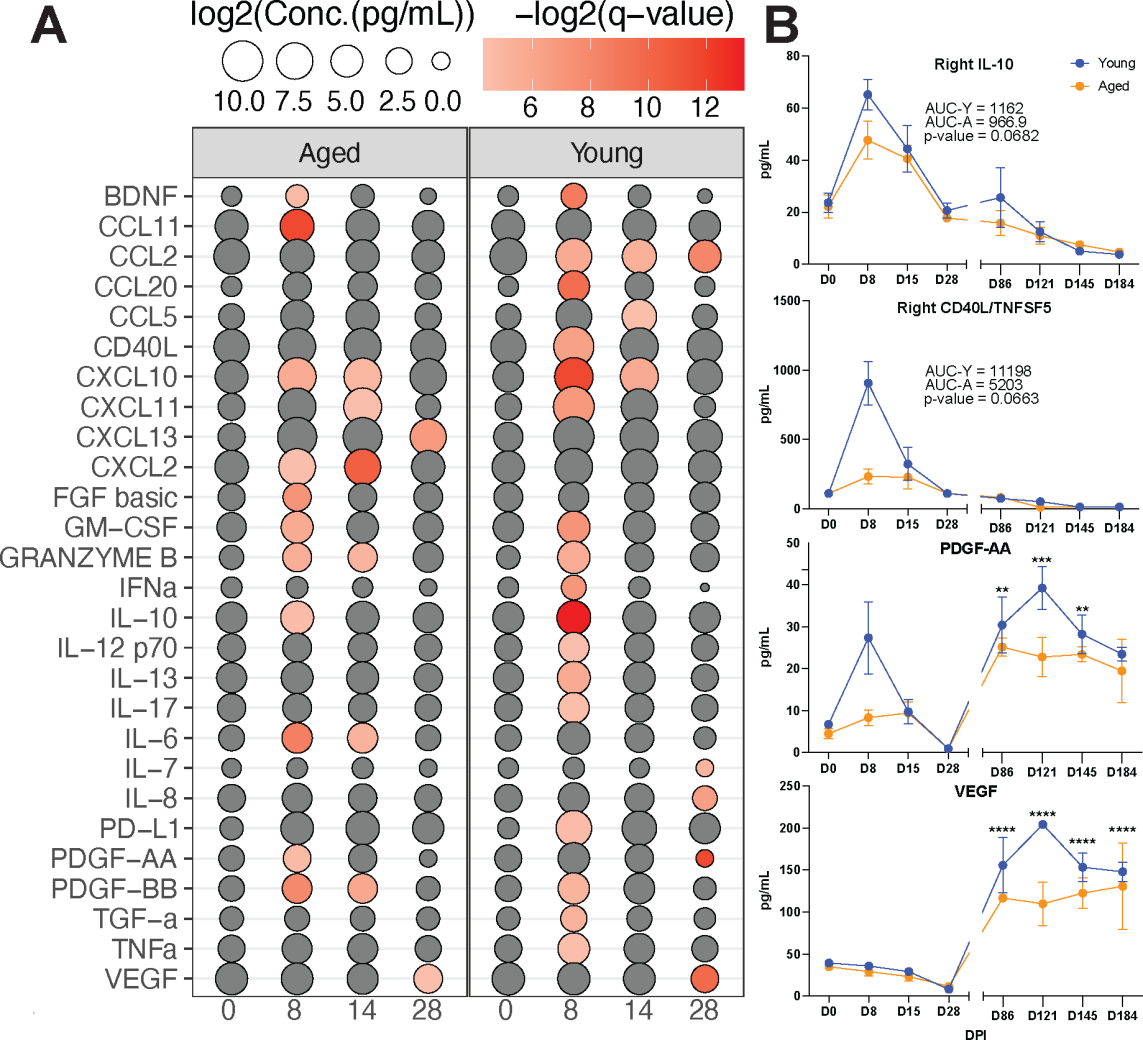
A robust inflammatory response only in the infected lung. (**A**) Bubble plot of the upregulated immune mediators in BAL supernatants determined by Luminex assays at the indicated time points in the right lungs of young and aged macaques. The size of the bubble represents the concentration of the immune mediator, while the color represents the significance of the change. (**B**) Line graphs of IL-10, CD40L, PDGF-AA, and VEGF production. Each time point from all animals was compared to 0 DPI, and AUC was determined for both young and aged groups and compared to each other. *P < 0.05, **P < 0.01, and ***P < 0.001.

Immune mediator production in the right and left BAL was assessed longitudinally using Luminex to better understand the immune response at the beginning and end of the infection. A significant increase in the concentration of numerous cytokines and chemokines was at detected 8–28 DPI in only the right but not the left BAL (data not shown) of both young and aged animals (Fig. 4A). An area under the curve (AUC) analysis revealed that IL-10 (*P* = 0.0682) (anti-inflammatory) and CD40L (*P* = 0.0663) (co-stimulatory) levels were modestly higher in young compared to aged animals (Fig. 4B). Increased levels of lymphocyte and neutrophil chemokines (CCL11 and CXCL2), growth factors (FGF basic and PDGF-AA), and IL-6 (acute phase response) were only detected in BAL from aged animals. On the other hand, lymphocyte and granulocyte chemokines (CCL20 and CCL5), Th1 cytokines (IL-12 and TNFα), growth factors (TGFα), CD40L (costimulatory), PD-L1 (inhibitory), and IL-13 (mucus secretion) were only significantly increased in BAL from young animals. For both young and aged animals, most immune mediators peaked at 8 DPI with some exceptions. CXCL-10 (T-cell attractant) levels remained high at 14 DPI. The levels of CXCL2 (neutrophil chemoattractant), Granzyme B (cytotoxicity), IL-6, and PDGF-BB (tissue repair) were increased at 8 and 14 DPI in aged animals. Increased B-cell chemoattractant CXCL13 was only observed in aged animals at 28 DPI. Vascular endothelial growth factor (VEGF) (angiogenesis and tissue repair) levels were significantly decreased at 28 DPI for both age groups. CCL2 (granulocyte chemoattractant) levels were decreased in the young at 8–28 DPI, while IL-7 (lymphocyte homeostasis), IL-8 (neutrophil chemoattractant), and PDGF-AA (tissue repair) levels decreased only in young animals at 28 DPI (Fig. 4A). Most of these immune mediators remained low, except for PDGF-AA and VEGF, which were significantly increased at 89–184 DPI (Fig. 4B).

### MAH infection induces shifts in the immune composition of BAL

**Fig 5:**
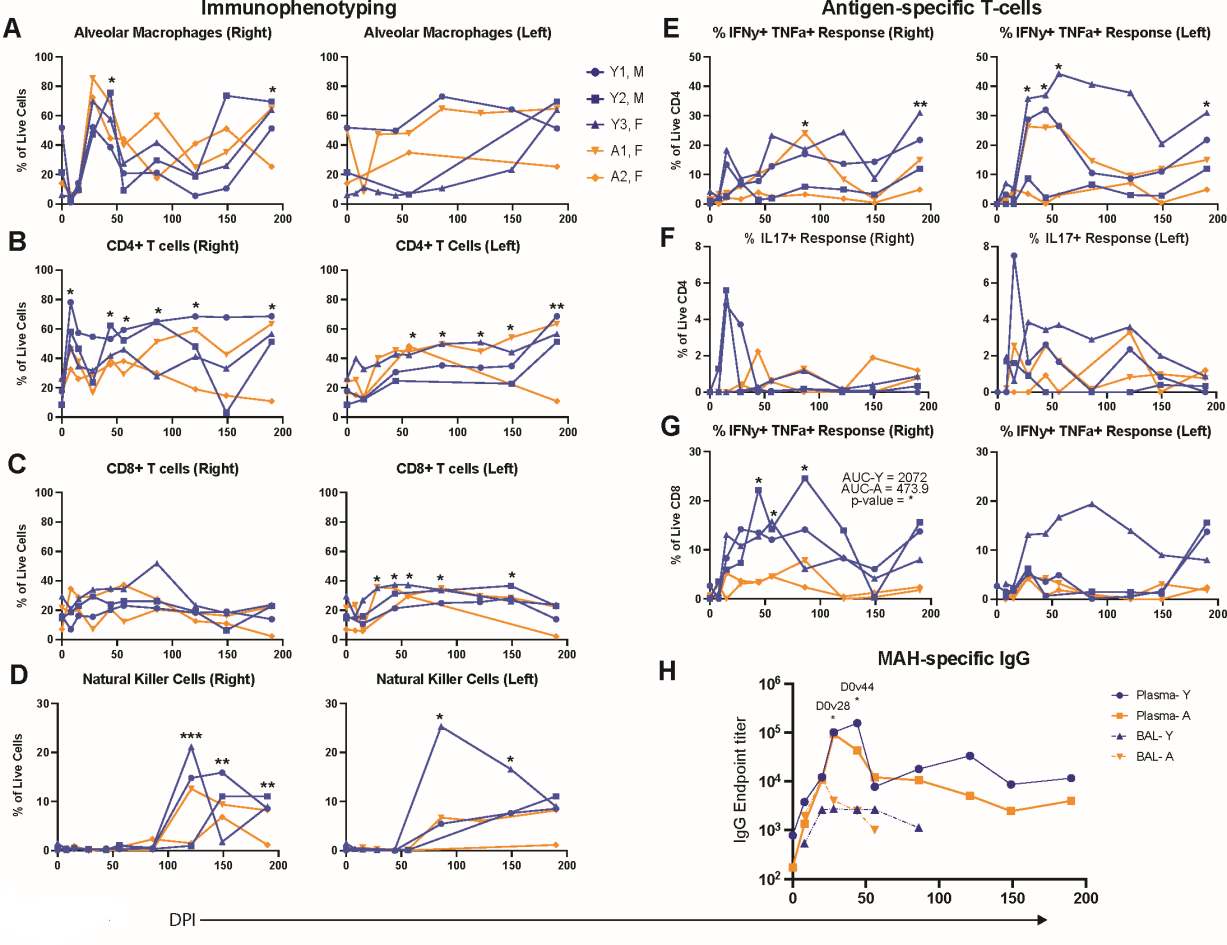
Immune infiltrates observed in both lungs. BAL samples were collected from the right and left lungs at various days post-infection, and flow cytometry data were acquired. The percent abundances of live (**A**) alveolar macrophages, (**B**) CD4+ T cells, (**C**) CD8+ T cells, and (**D**) NK cells are shown. The MAH-specific CD4 and CD8 T-cell response was determined by intracellular cytokine staining for IFNγ, TNFα, and IL-17 following stimulation with 1 mg/mL of MAH lysate. The percent responses of (**E**) IFNγ+ TNFα+ CD4 T cells, (**F**) IL-17+ CD4 T cells, and (**G**) IFNγ+ TNFα+ CD8 T cells are shown. (**H**) MAH-specific IgG endpoint titer averages in the plasma and BAL of the young and aged animals. For panels **A–H**, each time point from all animals was compared to 0 DPI, and AUC was determined for both young and aged groups and compared to each other. *P < 0.05, **P < 0.01, and ***P < 0.001.

Flow cytometry was used to assess the immune response to MAH (Fig. S3A through C). Prior to infection, AMs were the most abundant immune cells in the BAL. However, the relative frequency declined in the right lung at 8–14 DPI before rebounding to pre-infection levels at 44 DPI and fluctuating for the remainder of the study (Fig. 5A). The decrease in AM frequency was coupled with a significant influx of CD4 T cells at 8 DPI (Fig. 5B), while frequencies of CD8 T cells remained unchanged (Fig. 5C). The frequency of CD4 and CD8 T cells remained higher compared to 0 DPI in the left lung (Fig. 5B and C). The frequency of natural killer (NK) cells increased in both lungs for the aged group at the later stages of infection (Fig. 5D). Antigen-specific CD4 and CD8 T-cell responses were sporadically detected at 28–190 DPI due to large animal-to-animal variations (Fig. 5E through G). Two young animals generated detectable IL-17+ CD4 T-cell responses in both the right and left BALs (Fig. 5F). The frequency of MAH-specific CD8 T cells was higher in young animals in the right BAL (Fig. 5G).

Additionally, we performed an ELISA assay on plasma and bilateral BAL supernatants to measure the systemic and local MAH-specific IgG antibody titers, respectively. Plasma IgG levels showed a rapid rise from 0 to 44 DPI followed by a decline and plateau from 56 to 190 DPI (Fig. 5H). The BAL IgG profile exhibited a modest and transient increase from 8 to 14 DPI followed by a steady decline from 14 to 86 DPI, ultimately reaching nadir levels (Fig. 5H).

### Transcriptional changes in the lung immune landscape over the course of infection

**Fig 6:**
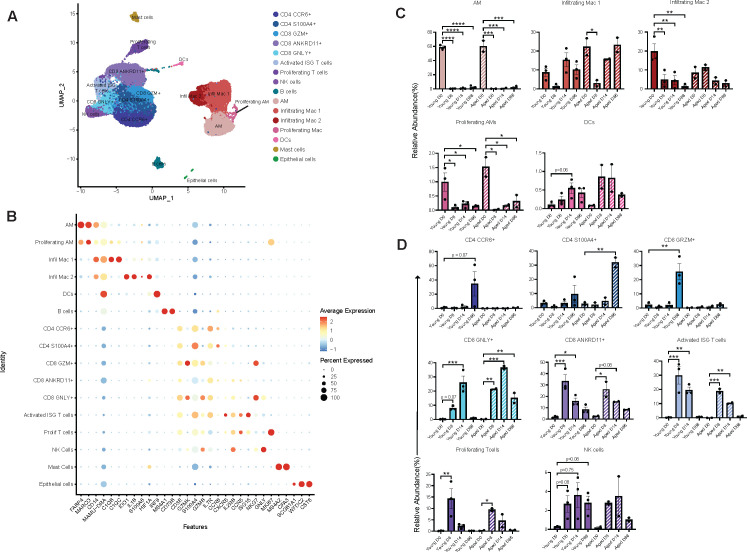
Single-cell RNA sequencing analysis reveals shifts in the immune landscape of the lungs. (**A**) Uniform manifold approximation and projection (UMAP) representation of 79,154 immune cells within four time points across MAH inoculation (days 0, 8, 14, and 86) showing 16 unique clusters. (**B**) Bubble plot of key gene markers used to annotate the UMAP. (**C**) Significant relative abundances of myeloid clusters by DPI. (**D**) Significant relative abundances of lymphoid clusters by DPI. Significance for panels C and D was calculated using a one-way ANOVA with Benjamini and Hochberg’s *post hoc* FDR comparisons relative to 0 DPI. *P < 0.05, **P < 0.01, ***P < 0.001, and ****P < 0.0001.

Next, we longitudinally profiled BAL cells (0, 8, 14, and 86 DPI) using single-cell RNA sequencing. These time points were chosen to capture the transcriptome before (0 DPI), at the height (8 and 14 DPI), and midway through (86 DPI) the infection. Several distinct lymphoid and myeloid clusters were identified based on canonical markers and cells from both age groups, and all time points contributed to the clusters identified (Fig. 6A; Fig. S4A). AM subsets were identified based on the expression of *FABP4* and *MARCO*, while infiltrating macrophages expressed higher levels of *CD14* and various inflammatory markers, including *S100A8* and *IL1B*. Dendritic cells (DCs) expressed high levels of *MAMU-DRA* and *IRF8*. All T-cell clusters expressed varying levels of *CD3E* and were delineated into either CD4 or CD8 T cells based on the expression of *S100A4*, *CCR6* (CD4) and cytotoxicity molecules, *GRZMB*, *GNLY* (CD8). The NK cells expressed cytotoxic molecules (*GNLY* and *GRZM*) and NK cell receptor, *KLRD1*, in the absence of CD3. B cells exclusively expressed canonical markers *MS4A1*, *CD79B,* and *CD79A*. Finally, small clusters of proliferating AMs and T cells expressing *Ki67*, mast cells (*MS4A2*), and epithelial cells (*WFDC2* and *CST6*) were identified (Fig. 6B; Fig. S4B).

Cell type assignment was verified through functional enrichment of marker genes. Those defining AM subsets enriched to gene ontology (GO) terms associated with response to oxidative damage and anti-microbial defense, while proliferating AM highly expressed cell cycle genes (Fig. S4C). Infiltrating macrophages expressed genes important for wound healing, chemotaxis, phagocytosis, and inflammation (Fig. S4C). In contrast, marker genes that define DC play a role in endocytosis and regulation of the defense response (Fig. S4C). Markers of CD4 clusters enriched to GO terms associated with helper T cells (regulation of immune effector process and cytokine production) (Fig. S4D). Marker genes of CD8 subsets enriched to GO terms “cytolysis,” “alpha-beta T-cell activation,” and “response to virus” (Fig. S4D). The gene markers for NK cell clusters play critical roles in cytolysis and C-lectin receptor signaling (Fig. S4D). Mast cells expressed genes associated with inflammatory response and wound healing, while epithelial cells were enriched to cilium movement and organization (Fig. S4E).

We next determined changes in the relative abundance of these clusters throughout infection (Fig. 6C and D). Corroborating the flow cytometry data, the frequency of AM and infiltrating macrophages in both age groups dropped precipitously by 8 DPI. While the frequency of AM and infiltrating macrophage 2 (IM2) subsets remained low, that of infiltrating macrophage 1 (IM1) in young and aged animals rebounded. Frequency of DCs showed a modest increase at 14 DPI only in young animals (Fig. 6C). Frequencies of all T-cell subsets increased following infection in a subset, age, and DPI-dependent manner (Fig. 6D). Notably, the frequency of CD4 CCR6+ and CD8 GRZM+ T cells increased only in young animals at 86 DPI, while that of CD4 S100A4+ cells significantly increased at 86 DPI in aged animals only. CD8 GNLY+ increased at 8 DPI peaking at 14 DPI in both age groups, while that of the remaining T-cell subsets peaked at 8 DPI and remained high at 14 DPI (Fig. 6D).

### Aging dysregulates transcriptional response to MAH infection

**Fig 7:**
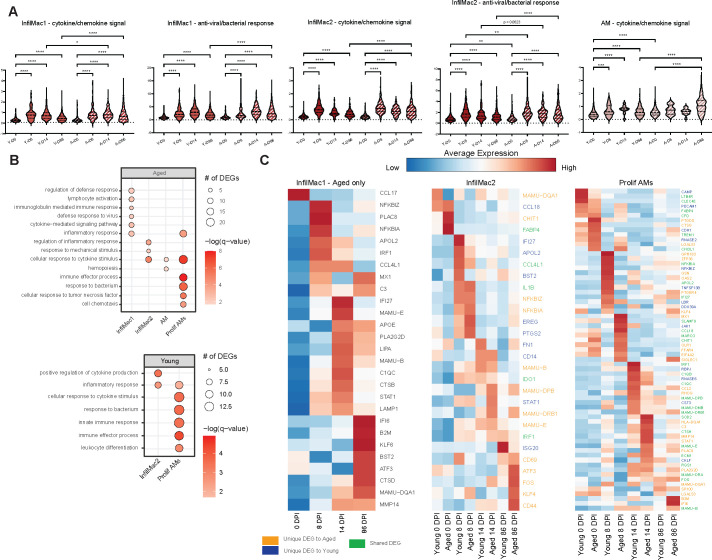
Age-mediated macrophage transcriptional response to MAH infection. (**A**) Violin plots of module scores within macrophage clusters from young and aged animals across DPI. (**B**) Bubble plots of the GO terms to which differentially expressed genes (DEGs) within macrophage clusters from young or aged animals identified using PALMO enriched. The size of the bubble indicates the number of differentially expressed genes that enriched to their respective GO term, and the −log(*q*-value) is indicated by color. (**C**) Heatmap of significant DEGs within macrophage clusters of young or aged animals. Scaled average gene expression is represented by the color within each heatmap from low (blue) to high (red). DEGs are colored based on whether they are unique to aged (orange), young (blue), or shared between the two groups (green). Significance for panel A was calculated using a Kruskal-Wallis one-way ANOVA with Benjamini and Hochberg’s *post hoc* FDR comparisons relative to 0 DPI. *P < 0.05, **P < 0.01, ***P < 0.001, and ****P < 0.0001.

In addition to assessing changes in subset frequency, we investigated longitudinal gene expression changes within key clusters. Module scores of gene sets important for “cytokine-chemokine signaling” and “anti-viral/bacterial responses” previously described in references (40, 41) significantly increased at 8–86 DPI in several of the macrophage subsets regardless of age (Fig. 7A). Interestingly, all the module scores at 86 DPI were higher in aged compared to young animals, indicating a heightened myeloid response at later stages of the disease (Fig. 7A).

We also investigated longitudinal changes in gene expression using the Platform for Analyzing Longitudinal Multi-omics data (PALMO) (42). A larger number of differentially expressed genes (DEGs) were detected in the infiltrating macrophage subsets from aged animals, which enriched to pathways such as “cytokine-mediated signaling pathways,” “defense response to virus,” “inflammatory response,” and “regulation of defense response” (Fig. 7B). In the IM1 subset in aged animals, the expression of inflammatory and interferon-stimulated genes (ISG) was significantly increased at 8 and 14 DPI (Fig. 7C). DEGs within IM2 from young animals also enriched to “positive regulation of cytokine production” and “inflammatory response” (Fig. 7B). However, despite similar functional enrichment, each age group exhibited a unique transcriptional signature (Fig. 7C). Similarly, DEG detected in the proliferating AM subsets from young and aged animals enriched to gene ontology terms associated with innate immunity and response to cytokines, but the number of shared DEGs was limited (Fig. 7B and C).

**Fig 8:**
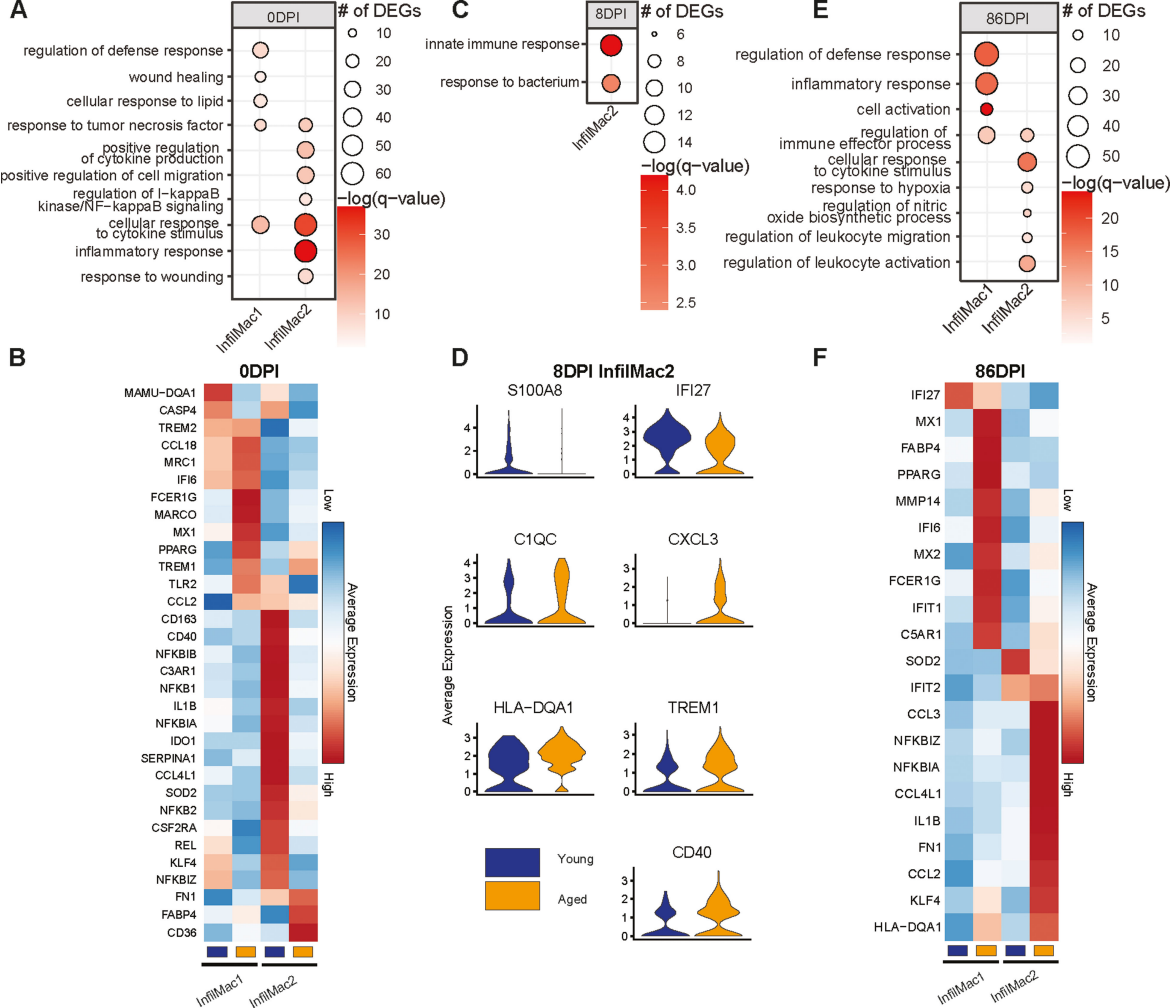
Aging leads to a dysregulated infiltrating macrophage response to MAH infection. Bubble plots of the GO terms to which DEGs were identified using EgdeR-QLF within infiltrating macrophage clusters comparing young and aged animals enriched at (**A**) 0, (**C**) 8, and (**E**) 86 DPI. The size of the bubble indicates the number of genes that enriched to that GO term, and color indicates the significance compared to young animals. Heatmap and violin plots of DEGs within infiltrating macrophage clusters at (**B**) 0, (**D**) 8, and (**F**) 86 DPI. Scaled average gene expression is represented by the color within each heatmap from low (blue) to high (red).

We next used EdgeR QLF to uncover DEG between young and aged animals at each DPI (43) (Fig. 8). DEGs were detected in IM subsets prior to infection, enriching pathways such as “regulation of defense response,” “cellular response to lipid,” “cellular response to cytokine stimulus,” and “inflammatory response” (Fig. 8A). DEGs downregulated with age at 0 DPI were involved in antigen presentation (*MAMU-DQA1*), apoptosis (*CASP4*), NFκB signaling (*NFKBIB, NFKB1, NFKBIA, NFKB2,* and *NFKBIZ*), and inflammatory response (*MX1, IL1B,* and *IDO1*). On the other hand, DEGs upregulated with age played a role in signaling (*TREM1, TREM2,* and *TLR2*), chemotaxis (*CCL18* and *CCL2*), and senescence (*FN1*) (Fig. 8B). DEGs within IM subsets were also noted at 8 DPI, enriching to GO terms such as “innate immune response” and “response to bacterium” (Fig. 8C). Increased gene expression of alarmin (e.g., *S100A8*) and ISG (e.g., *IFI27*) was detected in the young animals, while the expression of *C1QC, CXCL3, HLA-DQA1, TREM1,* and *CD40* was increased in aged animals at 8 DPI (Fig. 8D). DEGs at 86 DPI enriched to “responses to inflammation,” “hypoxia,” “regulation of the defense response,” and “nitric oxide biosynthesis” (Fig. 8E). At 86 DPI, the expression of pro-inflammatory (*MX1, IFI6, MX2, IFIT1,* and *IL1B*), chemotactic (*CCL3* and *CCL2*), senescence (*FN1*), and NFκB signaling genes (*NFKBIZ* and *NFKBIA*) was increased in IM2 from aged animals (Fig. 8F).

Cytotoxicity module scores (40) in CD8 T cells were increased over the course of the infection in both young and aged animals (Fig. S5A). Therefore, we investigated longitudinal gene expression changes within lymphoid cells using PALMO (42). DEGs detected within the CD4 S100A4+ subset enriched to GO terms associated with cytokine responses, while those detected in CD8 ANKDR11+ subset enriched to signaling, migration, and cytotoxicity (Fig. S5B). DEGs within ISG T cell cluster enriched to type 1 IFN signaling, while those in NK cells played a role in cytotoxicity (Fig. S5B). DEGs within the CD4 S100A4+ T-cell subset detected in aged animals included genes important for T cell receptor signaling (*CD3D, CD3E, CD3G, LCK,* and *JAK1*), activation marker *CD69*, and effector molecule *IFNG* (Fig. S5C). Similarly, increased expression of TCR signaling molecules, chemokine receptors, and ISG was detected in ANKRD11+ (*ZAP70, CD2, FOS, CXCR4, CCR5, ISG15*, and *STAT1*) (Fig. S5C). Smaller overlap in DEGs was detected in the remaining T-cell clusters with more robust gene expression changes detected in NK cells from aged animals with upregulation in several ISGs (*ISG15, IRF1,* and *STAT1*) and granzymes (*GZMB, GZMA,* and *GZMK*) (Fig. S5C).

### MAH infections cause decompartmentalization of the lung microbiome

**Fig 9:**
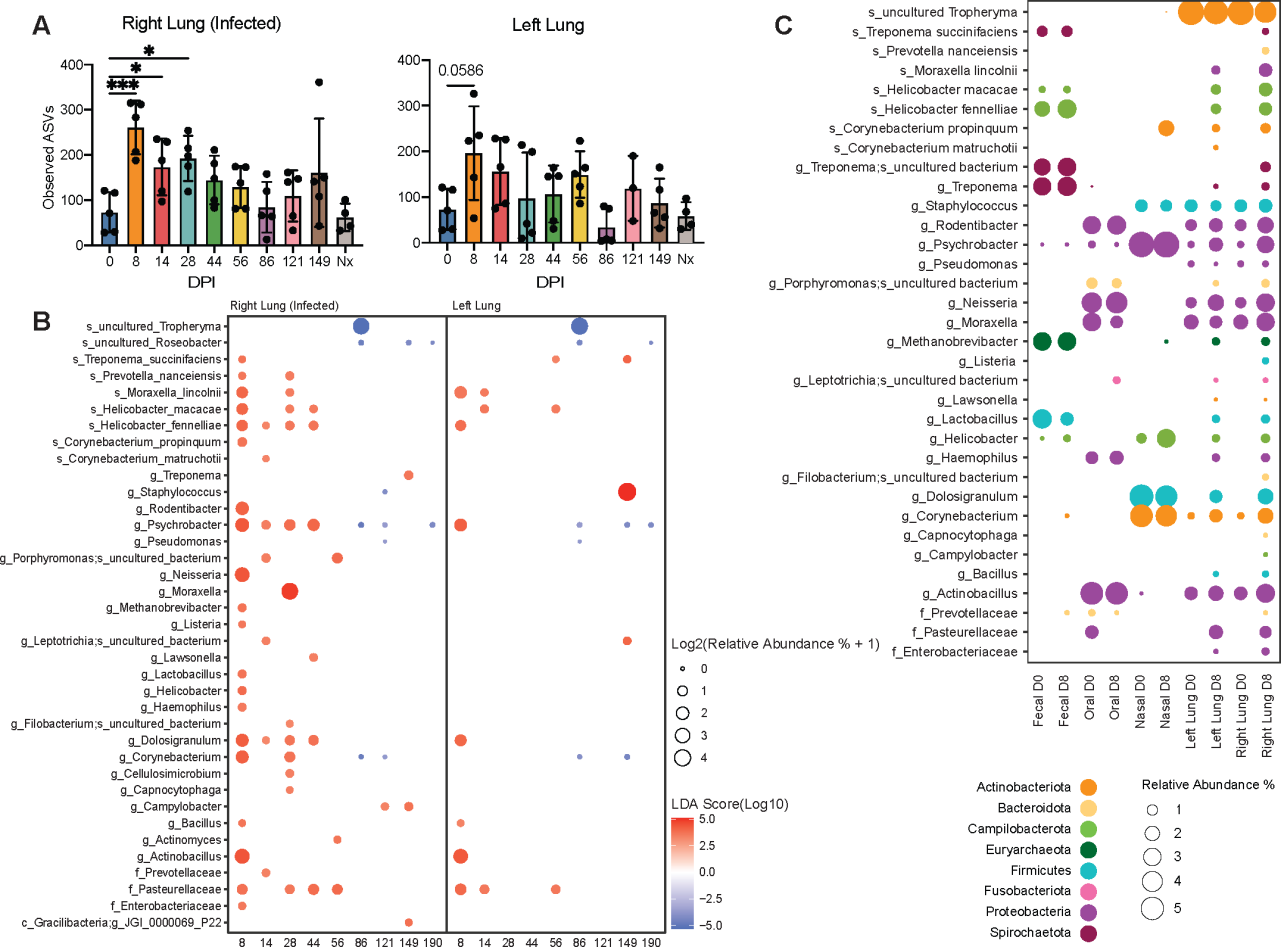
Microbiome profiling reveals microbial decompartmentalization of the lower respiratory tract. (**A**) Observed amplicon sequence variants in the right and left BAL longitudinally. (**B**) Bubble plot derived from linear discriminant effect size analysis of the microbiome profiling of the right and left BAL. The size of each bubble represents the percent relative abundance, and color represents the linear discriminant analysis (LDA) score. (**C**) Bubble plot of the most abundant species, family, or genera at each location at days 0 and 8. The size of each bubble represents the percent relative abundance, and color represents the phyla.

We profiled the BAL, nasal, oral, and gut microbiomes using 16S rRNA gene amplicon sequencing longitudinally. Overall, BAL communities were intermingled with oral and nasal communities, while the fecal microbial communities were distinct (Fig. S6A). The fecal community was dominated by Spirochaetota (*Treponema*), Firmicutes (*Lactobacillus* and *Ruminococcus*), and Bacteroidota (*Alloprevotella*), while the nasal, oral, and lung communities were dominated by Proteobacteria (Fig. S6B and C). *Tropheryma* was uniquely found in the lung with lower abundance in the aged group as recently reported (39) (Fig. S6C). Following infection, the number of observed amplicon sequence variants (ASVs) was significantly increased in the right BAL of both young and aged animals at 8–28 DPI (Fig. 9A). Additionally, beta diversity was decreased in the right BAL early in infection as evident by the diminished UniFrac distance at 8 DPI (Table S2). Despite the lack of evidence of MAH spread to the left lung, a modest increase in the number of observed ASVs was detected at 8 DPI (Fig. 9A). Diversity of the nasal, oral, and fecal microbiomes was unaffected by MAH infection (Fig. S6D).

Linear discriminant effect size (LEfSe) analysis was carried out longitudinally, revealing an increase in the relative abundance of ASVs typically found in fecal (*Helicobacter*, *Treponema*, *Methanobrevibacter*, and *Lactobacillus*), oral (*Haemophilus*), and nasal (*Corynebacterium*, *Dolosigranulum*, and *Psychrobacter*) communities within the right lung at 8–56 DPI (Fig. 9B and C). ASVs of species that typically dominate the lung microbiome decreased in abundance at 86–190 DPI (*Tropheryma* and *Roseobacter*) (Fig. 9B). Shifts in microbial communities were less dramatic in the left lung; nevertheless, increased abundance of *Actinobacillus, Moraxella lincolnii,* and *Helicobacter fennelliae* were noted at 8–14 DPI (Fig. 9B).

## DISCUSSION

Our group has previously reported pulmonary disease in one out of three middle-aged, bilaterally oophorectomized female rhesus macaques after MAH intrabronchial infection (36). The affected animal exhibited bronchiectasis, infiltration of immune cells into the lung, and granuloma formation. In this study, we extended these earlier observations to interrogate the impact of age on the pathophysiology of acute pulmonary MAH infection. Radiological findings showed comparable inflammatory changes during the acute phase of the infection in young and aged animals followed by resolution and then recrudescence of inflammatory changes in aged animals only. At necropsy, pulmonary consolidation and lesions were detected only in aged animals. Histological analyses revealed the presence of lymphoid aggregates, interstitial fibrosis, pulmonary edema, and bronchopneumonia in aged animals. Surprisingly, granulomatous disease was absent in these aged animals. One possible explanation is that, in contrast to the prior report, the animals in this study were not oophorectomized, and epidemiological findings suggest a higher incidence of NTMPD in postmenopausal females (1, 6, 44). However, the spectrum of radiologic changes is reminiscent of the heterogeneity in NTMPD radiologic changes seen in humans (45).

Interestingly, MAH could only be detected by culture at 8 DPI, and no age-related differences in bacterial burden in BAL were observed by qPCR. Our inability to detect live organisms beyond 8 DPI in the BAL could be due to the robust inflammatory response that developed at 8 and 14 DPI. This is especially relevant for MAH, a slow-growing mycobacterium. However, biofilms containing MAH were found in the trachea of A2 at necropsy. This observation suggests that MAH was able to persist in sites that are not adequately sampled by BAL procedure such as epithelial cells (46–49). The persistence of MAH may explain the worsened pathology observed in aged animals at the late stages of the study causing prolonged immune activation as revealed by the scRNA-seq data. Investigating the epithelial cells of the lung for the presence of MAH will be essential in future studies.

In line with the minimal bacterial dissemination from the original site of infection to the left lung, significant changes in soluble immune mediators were only observed on the right side. These changes show increased levels of T cell, macrophage, eosinophil, DC, and B-cell chemoattractants that correlated with the infiltration of these cells into the alveolar space and lung parenchyma, revealed by histology at necropsy. Growth factors that play a critical role in wound healing (VEGF and PDGF-BB) were elevated later in the disease process in line with the initiation of the post-infectious repair phase. Interestingly, the levels of PD-L1 were only significantly increased in young animals, while IL-10 and CD40L were modestly lower in aged animals, indicating that increased lung damage with age may be due in part to a heightened inflammatory response (50) and reduced co-stimulation (51). Additionally, the levels of IL-12 and TNFα were only significantly increased in the BAL of young animals 8 DPI, suggesting a more robust Th1 response in young animals (52).

Initially, the relative abundance of AMs precipitously declined due to the influx of T cells. Although we identified a cluster of proliferating AMs, in line with studies showing reconstitution of AMs after inflammation-related depletion in mice and humans (53, 54), AM levels did not recover to pre-infection levels. Rather, they were replaced by infiltrating monocyte-derived macrophages (IMs), likely recruited through GM-CSF. RNA sequencing suggests that IMs from aged animals may be functionally different than those of young animals before the infection in line with our prior studies (39). Additionally, differential gene expression revealed heightened inflammatory signatures in macrophages of old animals in response to infection, including greater and more prolonged induction of MHC-II molecules, ISGs, complement, and cathepsins. These data are consistent with an inability to regulate inflammatory responses in aged monocyte-derived macrophages (16).

The relative abundance of NK cells significantly increased during the late stages of infection in all animals. NK cells can activate MAH-infected macrophages to kill the pathogen via the secretion of TNFα and IFNγ (55, 56). NK cells of aged animals showed increased expression of cytolytic genes after infection, suggesting the presence of undetected pockets of bacterial replication or dysregulated NK cell reactivity that may be contributing to the increased lung damage.

In both young and aged animals, a large influx of CD4 T cells was detected early, and their levels remained elevated throughout the course of infection in both lungs, in line with the reported role of CD4 T cells in fighting mycobacteria (57). This response was dominated by T cells secreting IFNγ and TNFα. Interestingly, two of the young animals generated an IL-17 response, and the levels of IL-17 were significantly increased in the BAL at 8 DPI only in young animals, suggesting that a Th17 response may have been induced in the young lung. A larger MAH-specific CD8 T-cell response was detected in the right lung of young animals. High levels of MAH-specific IgG levels were detected at 14 DPI in the plasma followed by a decrease after 44 DPI, which correlates with the last time point MAH DNA was detected by qPCR in the BAL. The transient increase in plasma IgG may be due to the ability of mycobacteria to evade and subvert antigen presentation on MHC class II molecules (58–61). MAH-specific IgG levels in the BAL showed a modest transient increase, suggesting that IgG may be important in limiting dissemination. The role of IgG in limiting disease within the lungs may be complemented by surfactant proteins A and D, which can opsonize mycobacteria (62). Our data differ from studies showing that NTM infections result in high levels of circulating IgG and IgA, which can be used to diagnose NTM infection (63, 64). This difference could be due to the longer chronicity of disease in humans.

T-cell clusters expressing ISGs, which are crucial to combat mycobacteria (65), peaked during the acute phase, the same time levels of IFNα peaked in the BAL. Although a significant increase in IFNα was only detected in young animals at 8 DPI, robust induction of ISGs was also evident in aged animals. CD8 T cells expressing high levels of *GNLY*, which plays a role in clearing mycobacteria (66), and *ANKRD11* peaked as well. Two different CD4 T-cell clusters expressing high levels of *CCR6+* and *S100A4+* appeared late post-infection in young and aged animals, respectively. CCR6+ CD4 T cells were recently implicated in the prevention of tuberculosis-associated immune reconstitution syndrome, and their detection only in young animals aligns with better disease resolution in this group (67). Alarmins are known to generate an immune response by acting as damage-associated molecular patterns (68–70); therefore, the increased presence of CD4+ T cells expressing alarmins (*S100A4*) in aged animals aligns with the notion of a dysregulated inflammatory response in this group.

Significant disruption in the BAL microbiome was noted in both the right and left lungs that were sustained beyond peak bacterial load. Disease states such as cystic fibrosis or bronchiectasis often cause dysregulation of the normal lung microbiota, characterized by increased microbial biomass, but lower diversity (24). In contrast, our data show that MAH infection leads to increased diversity in the microbial community of the lungs, which is accompanied by the detection of several species normally sequestered in other anatomical sites. It remains unclear whether these data indicate the movement of live organisms or bacterial DNA fragments due to the impaired barrier. Regardless, bacterial products can activate myeloid cells, exacerbating inflammatory responses and further damaging tissues (71). This could explain the sustained activation of AMs and IMs 3 months after infection. Additionally, a reduction in the relative abundance of *Tropheryma,* a major component of a healthy rhesus macaque lung microbiome (39), was observed after infection. Collectively, our data suggest that decompartmentalization of microbial communities and dysregulation of both the T-cell and macrophage response to infection are associated with increased disease severity.

Limitations of the study include the small cohort size, the use of only aged females, and the administration of a single bolus of MAH in only the right caudal lobe. Future studies would benefit from examining a larger cohort that addresses sex as a biological variable and instilling MAH with an inhalation chamber to closely recapitulate human exposure. Nevertheless, this study successfully established a rhesus macaque model of MAH pulmonary disease that recapitulates age-associated increased pulmonary disease severity in humans. This model addresses the limitation of rodent models where NTM inoculation results in disseminated, and not pulmonary, disease and clinical studies where ill-defined infection timelines, a lack of healthy controls, and hard-to-control variables (smoking history, living conditions, antibiotic use, etc.) confound outcomes. The availability of this model will facilitate future studies that interrogate host-pathogen interactions and the development and testing of novel therapeutics.

## MATERIALS AND METHODS

### Cohort description, animal infection, and sample collection

Three young (5–10 years; two females, one male) and two aged (>18 years; two females) rhesus macaques (*Macaca mulatta*) were intrabronchially inoculated in the right caudal lobe with 6.8 × 10^8^ CFU of MAH (Table S1). BAL and oral, nasal, and fecal swabs were collected at 0, 8, 14, 28, 44, 56, 86, 121, 149, and 190 days post-infection. Animals underwent thoracic CT scanning with controlled lung inflation with a GE Optima CT 660 prior to MAH inoculation and on 13, 41, 83, 118, 146, and 183 DPI. The severity of radiologic changes was evaluated by a blinded radiologist and assigned a severity score based on the appearance of radiologic abnormalities, including ground glass, mucus, volume loss, lymphadenopathy, detection and severity of bronchiectasis, presence and size of cavitations, scarring, and tree in bud opacity with a score of 1 being minimal findings to 6 being severe. Data were generated bilaterally at select time points for each experimental parameter (Table S3).

### *Mycobacterium avium* stock

MAH was obtained from a patient with AIDS and shown to be virulent in mouse models (25). The bacteria were grown on Middlebrook 7H10 agar. The viability of the inoculum was 94% ± 2% determined as previously described (25).

### Bacterial culture and load

Bacterial load was determined by enumerating the number of colony-forming units in BAL supernatant after 8 weeks of incubation at 37°C/5% CO_2_ onto Lowenstein-Jensen agar plates. DNA was obtained from individual colonies, and the 16S rRNA region was amplified using forward and reverse primers (16S V4 region forward primer: CTGGCTCAGGATGAACGCT; reverse: GGGTCCCCGTCAATTCCTTT) (Invitrogen, Waltham, MA, USA) with the following conditions: 59°C annealing temperature, 60 s extension time, and 39 total cycles. Amplified DNA was run on agarose gel and extracted for sequencing.

For real-time quantitative PCR bacterial quantification, DNA was extracted from BAL supernatant using the DNeasy PowerSoil Pro Kit (Qiagen, Germantown, MD, USA) according to the manufacturer’s instructions. The bacterial burden of MAH was determined by qPCR using TaqMan Universal PCR Master Mix (Thermo Fisher Scientific, Waltham, MA, USA) and primers/probes specific for the IS1311 insertion sequence found in *M. avium* (72). Each run was initiated at 50°C for 2 min, then 95°C for 10 min, followed by 40 cycles at 95°C for 15 s, then 60°C for 1 min using a QuantStudio 3 Real-Time PCR System (Thermo Fisher Scientific). Extracted MAH DNA was used as the quantification standard.

### Electron microscopy

Swabs of the airways and intra-airway plug at necropsy were washed twice, and the pellet was suspended in a fixative buffer with 2.5% glutaraldehyde, 1% formaldehyde, and 0.1 M sodium cacodylate. Sections were stained, dehydrated, and visualized in the EM facility of Oregon State University as previously described (73). DNA was extracted from samples that were positive for biofilm or an intrabronchial plug with viable bacteria inside macrophages and subjected to PCR amplification using the primers and conditions described above.

### Flow cytometry

10^6^ BAL cells were surface stained with antibodies against CD4, CD20, CD27, IgD (Biolegend, San Diego, CA, USA), CCR7 (BD Biosciences, Franklin Lakes, NJ, USA), CD8b (Beckman Coulter, Brea, CA, USA), and CD28 (Tonbo Biosciences, San Diego, CA, USA). Cells were then fixed and permeabilized before the addition of anti-Ki67 (BD Biosciences, Franklin Lakes, NJ, USA). B cells (CD20) and T cells (CD8b and CD4) as well as naive and memory subsets were identified as previously described (39). 10^6^ BAL cells were stained with CD8a, CD14, CD16, CD123, CD206, HLA-DR (Biolegend, San Diego, CA, USA), CD11c (Invitrogen, Waltham, MA, USA), and Granzyme-B (BD Biosciences) to assess the frequency of innate immune cells (39). To measure the response of antigen-specific T cells, monocyte-derived macrophages, and DCs after MAH infection, 10^6^ BAL cells were stimulated with 1 mg/mL of MAH lysate in the presence of Brefeldin A for 16 hours. The cells were surface stained with CD8b (Beckman Coulter), CD4, CD14, CD20, and HLA-DR (Biolegend), then fixed and permeabilized followed by staining with IFNγ, IL-17 (Biolegend), and TNFα (Invitrogen). Data were acquired using the Attune NxT (Life Technologies, Carlsbad, CA, USA) and analyzed with FlowJo software (TreeStar, Ashland, OR, USA).

### Luminex

The levels of immune mediators in BAL supernatant were analyzed using the R&D 36-plex NHP XL Cytokine Premixed Kit (Bio-Techne, Minneapolis, MN, USA). Samples were acquired using the MAGPIX xMAP (Luminex Corporation, Austin, TX, USA). Data were analyzed using the Luminex Xponent software with an eight point logistic regression curve.

### Immunohistochemistry and immunofluorescence analysis

Five-micron thick sections of formalin-fixed paraffin-embedded macaque lung sections were placed onto glass slides and baked at 60°C for 1 hour and deparaffinized. Heat-induced antigen retrieval using the RNAScope Target Retrieval buffer (ACDBio Cat#322000) was then performed in a Biocare Decloaking Chamber at 110°C for 15 min. After cooling, the slides were washed in dH_2_O and TBS-T before overnight incubation with mouse anti-CD3 antibody (LN10 Biocare ACI B3152C) at 20°C. Slides were washed in 1× TBS-T, treated with 3% H_2_O_2_ in PBS for 10 min, and incubated with the Gold Bridge International Labs Polink 1 HRP Detection System against Mouse IgG (D12-110) (OriGene, Rockville, MD, USA) for 20 min at 20°C. CD3 staining was visualized with the CF488 tyramide fluorophore (Biotium 92171). The attached anti-CD3 antibody and Polink 1 secondary antibody were stripped off the tissue section by boiling in ACDBio Target Retrieval buffer for 15 min. The slides were incubated with a mixture of the goat anti-CD20 (Invitrogen PA1-9024), mouse anti-CD68/163 (CD68 Biocare CM033C; CD163 Thermo MA5-11458), and rabbit anti-MPO (Agilent A039829-2) antibodies overnight at 20°C. Slides were washed and incubated with secondary antibodies against mouse IgG (AF568, Thermo Fisher A10037), goat IgG (AF647, Thermo Fisher A21447), and rabbit IgG (Dy755, Thermo Fisher SA5-10043) for 2 hours at 20°C. The slides were counterstained with 4′,6-diamidino-2-phenylindole (DAPI) and cover slipped with the Prolong Gold mounting media (Thermo Fisher P36930). Stained slides were imaged using a Zeiss Axioscanner at 20×. Whole slide multispectral digital images were processed utilizing the HALO Image Analysis platform (v3.5.3577.214, Indica Labs, Albuquerque, NM, USA). Image analysis algorithms were constructed using the High-Plex FL module (v4.1.3). DAPI was utilized for nuclear segmentation, and unique thresholds for cytoplasmic positivity, as well as membranous and cytoplasmic positivity, were set for each antibody utilizing our previously established methods by author Allison, a board-certified pathologist with expertise in digital image analysis and immunofluorescence (74). To show that the signals are localized to the appropriate cell compartments, refer Fig. S2. Furthermore, Fig. S2 shows examples of signal quantification utilizing the image analysis module developed for this study, noting the absence of signal quantification without a well-visualized nuclear DAPI signal, eliminating non-specific signal counting.

### 16s amplicon sequencing and bioinformatics analysis

Amplification of the hypervariable V4 region of the 16s rRNA gene was performed using the 515F/806R PCR primers; the forward primers were conjugated with a 12-bp barcode (75). Each reaction was run in duplicate and prepared with GoTaq master mix (Promega Corporation, Madison, WI, USA). Cycling conditions were 94°C for 3 min, 37 cycles of 94°C for 45 s, 50°C for 1 min, and 72°C for 1 min, followed by a final cycle at 72°C for 10 min. The PCR products were multiplexed using Quant-iT PicoGreen dsDNA Assay Kits and dsDNA Reagents (Thermo Fisher). The resulting library was spiked with ~15%–20% PhiX and sequenced on an Illumina MiSeq. Raw FASTQ 16s rRNA gene amplicon sequences were processed using the QIIME2 analysis pipeline (76). Sequences were demultiplexed and filtered using DADA2 (77). MAFFT was used to align the sequence variants, while FastTree2 was utilized to construct a phylogenetic tree (78, 79). Taxonomy was assigned to sequence variants using q2-feature-classifier against the SILVA database (release 138) (80). The samples were rarified to 10,000 sequences per sample. QIIME2 was also used to generate alpha diversity metrics, while beta diversity was estimated using weighted and unweighted UniFrac distances (81). Differentially abundant bacteria between groups were identified via the LEfSe algorithm with a linear discriminant analysis score cutoff of 2 (82).

### Single-cell RNA library generation and bioinformatics analysis

Cryopreserved BAL cells from 0, 8, 14, and 86 DPI were thawed and then stained with Sytox orange (Thermo Fisher) to sort viable cells using an iCyt-Sony Cell Sorter System. Live cells were counted on a TC20 Automated Cell Counter (BioRad), tagged with cell multiplexing oligos, and pooled. The cells within each pool were diluted to a concentration of 1,600 cells/µL in ice-cold PBS with 0.04% BSA. Single-cell suspensions were loaded on the 10× Genomics Chromium Controller with a loading target of 20,000 cells. The libraries were prepared using the Chromium Single Cell 3′ Feature Barcoding Library Kit with the v3.1 chemistry (10× Genomics, Pleasanton, CA, USA). Libraries were sequenced using an Illumina NovaSeq with a target of 30,000 reads per cell RNA library and 2,000 reads per cell hashtag-oligo barcode library.

The reads were aligned and quantified using the Cell Ranger Single-Cell Software Suite (version 4.0, 10× Genomics) against the Mmul_8 rhesus macaque reference genome using the STAR aligner. Seurat (version 4.1.1) was used for downstream analysis of the aligned reads. The libraries were merged and cells with ambient RNA (<200 feature counts) and dying cells (>20% total mitochondrial gene expression) were filtered out (83). The resulting multiplexed library was further processed by performing variance stabilization with the SCTransform function, followed by data normalization using NormalizeData. The data were then scaled using ScaleData in preparation for PCA generation, which was accomplished using RunPCA. Thirty principal components were used to cluster the cells before uniform manifold approximation and projection (UMAP) generation using the FindNeighbors and FindClusters (resolution = 0.5) function in Seurat. UMAP generation was accomplished using runUMAP. Cell types were assigned to individual clusters using the FindMarkers function with a log2 fold change cutoff of 0.4. Differential expression analysis was performed using MAST with default settings in Seurat. All comparisons were performed between day 0 and days of notable DPI (8, 14, and 86). Gene expression changes were considered significant with a log fold change ≥ 0.58 and *P* ≤ 0.05.

PALMO ( v0.1.1) was used to determine longitudinal gene expression changes following MAH infection in both young and aged animals as described in Tutorial-6: Differential gene analysis in longitudinal data (42). Differentially expressed genes were significant with a log fold change ≥ 0.005 and corrected *P* ≤ 0.05. The Quasi Likelihood *F*-test in the Bioconductor EdgeR package (EdgeR QLF, v3.34.1) determined DEGs as previously described (43). DEGs were significant with a log2 fold change ≥ 0.58 and *P* ≤ 0.05.

### Statistical analysis

Statistical analysis was performed using GraphPad Prism software (GraphPad Software Inc., La Jolla, CA, USA). Statistical significance when comparing three groups or more was determined using a one-way ANOVA or a Kruskal-Wallis test with the Benjamini and Hochberg false discovery rate correction method for multiple comparisons. Significance when comparing two groups was measured using a two-tailed, unpaired parametric Welch’s or Student’s *t*-test.

## Data Availability

Complete data are available on the NCBI SRA with the following project accession numbers: PRJNA1010149 and PRJNA1006747.

## SUPPLEMENTAL MATERIAL

Figure S1 mbio.00829-24-s0001.tiff H&E stains, IF channels, IF stains, nuclear segmentation strategy, and quantification of immune cells.

Figure S2 mbio.00829-24-s0002.tiff Verification of HALO, the whole-slide image analysis platform.

Figure S3 mbio.00829-24-s0003.tiff Representative gating strategy for flow cytometry analysis.

Figure S4 mbio.00829-24-s0004.tiff Single-cell RNA sequencing by time point and age group.

Figure S5 mbio.00829-24-s0005.tiff The lymphocyte response to MAH infection is altered with age.

Figure S6 mbio.00829-24-s0006.tiff Microbiome profiling of the BAL samples and nasal, oral, and fecal swabs.

Table S1 mbio.00829-24-s0007.docx Metadata of the research animals.

Table S2 mbio.00829-24-s0008.docx The weighted intragroup Unifrac distances showing β-diversity at each time point and comparing to baseline (0DPI) for the right and left BAL.

Table S3 mbio.00829-24-s0009.docx Collection timeline of the various experimental readouts.

ASM does not own the copyrights to Supplemental Material that may be linked to, or accessed through, an article. The authors have granted ASM a non-exclusive, world-wide license to publish the Supplemental Material files. Please contact the corresponding author directly for reuse.
