# Supplementary figures and images for: Immunological and microbial shifts in the aging rhesus macaque lung during nontuberculous mycobacterial infection
Source: mBio. 2024 May 21;15(6):e00829-24. doi: 10.1128/mbio.00829-24 (PMC11237422; doi:10.1128/mbio.00829-24)

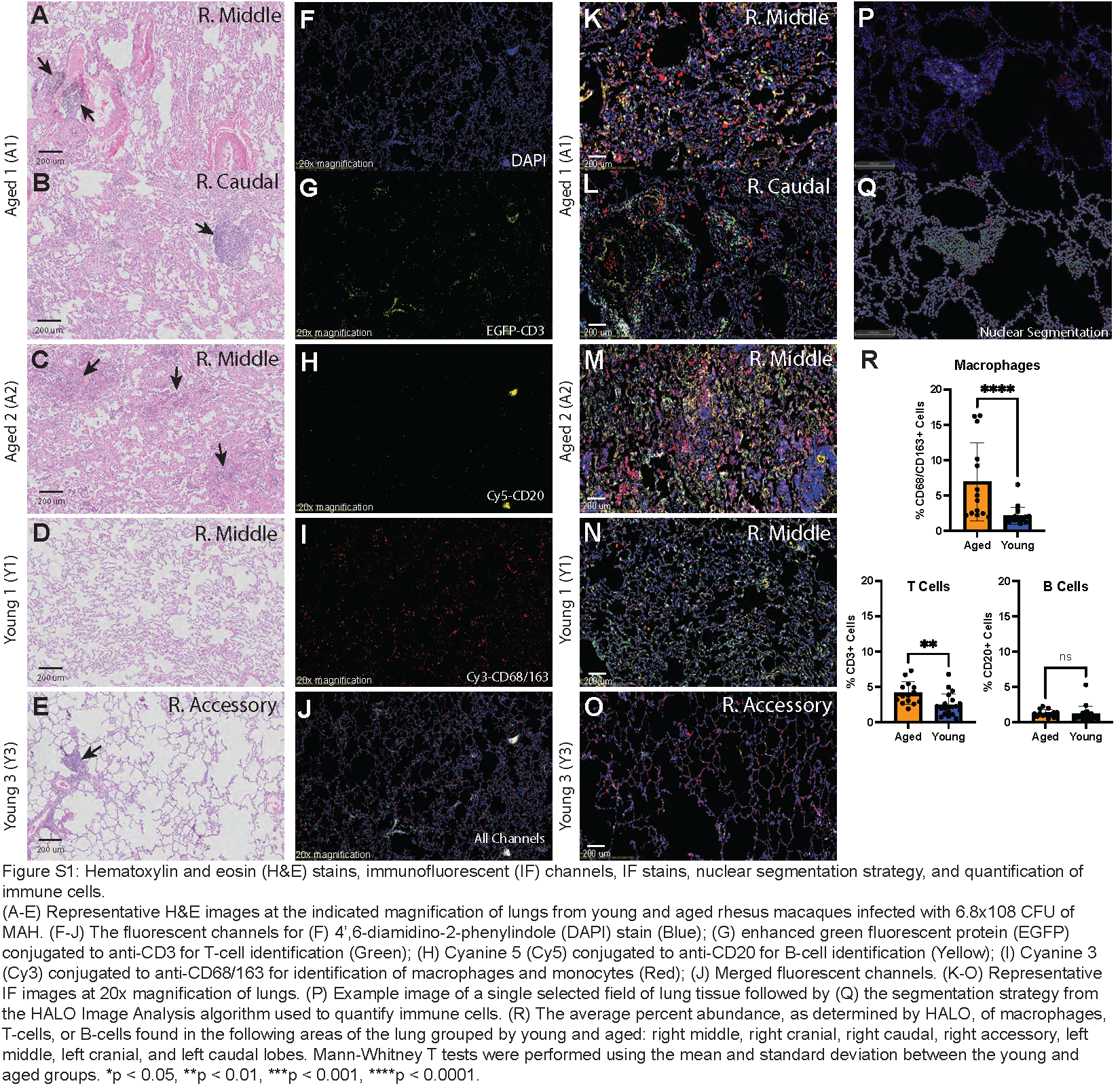

Supplement: Figure S1 — H&E stains, IF channels, IF stains, nuclear segmentation strategy, and quantification of immune cells. [file mbio.00829-24-s0001.tiff]

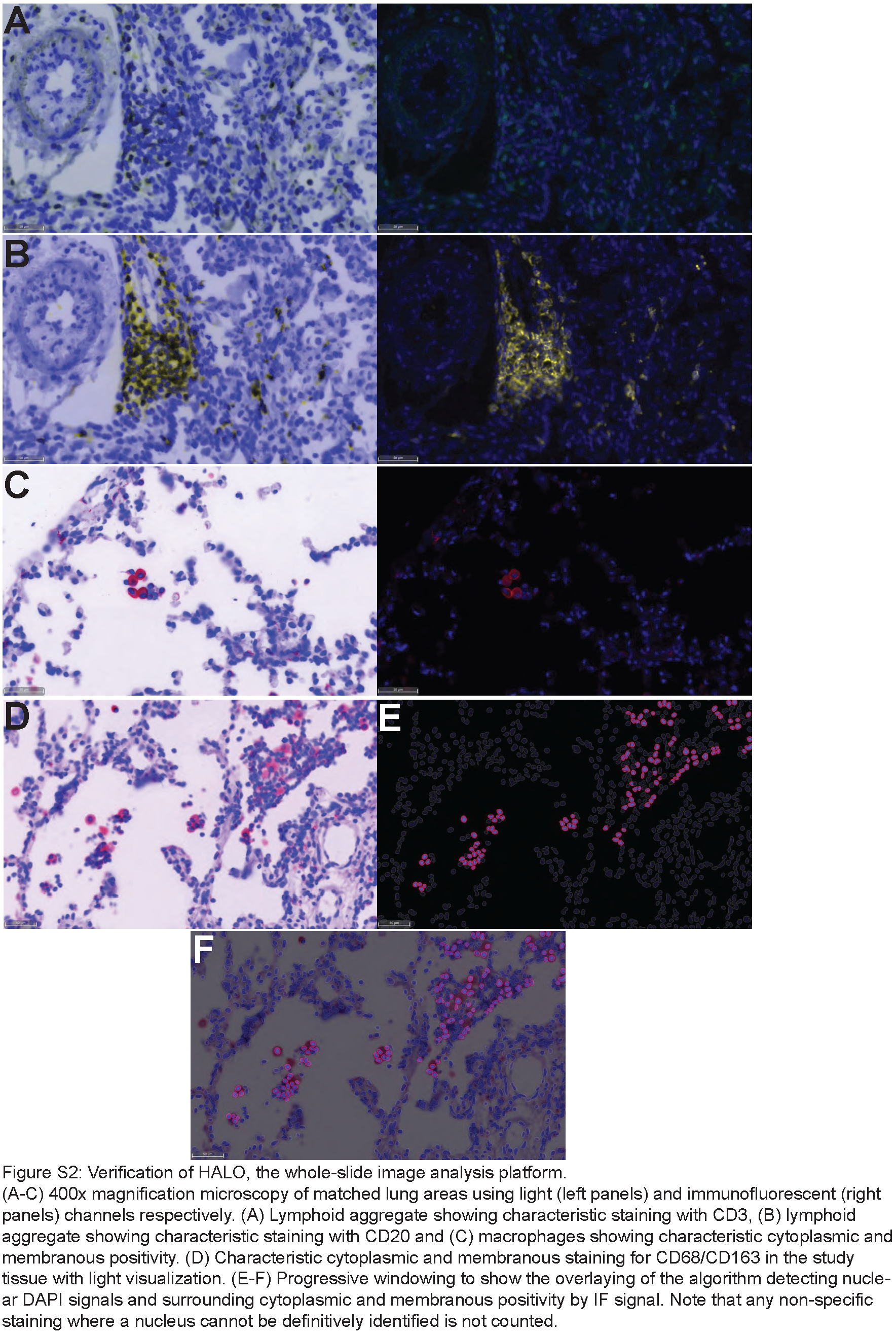

Supplement: Figure S2 — Verification of HALO, the whole-slide image analysis platform. [file mbio.00829-24-s0002.tiff]

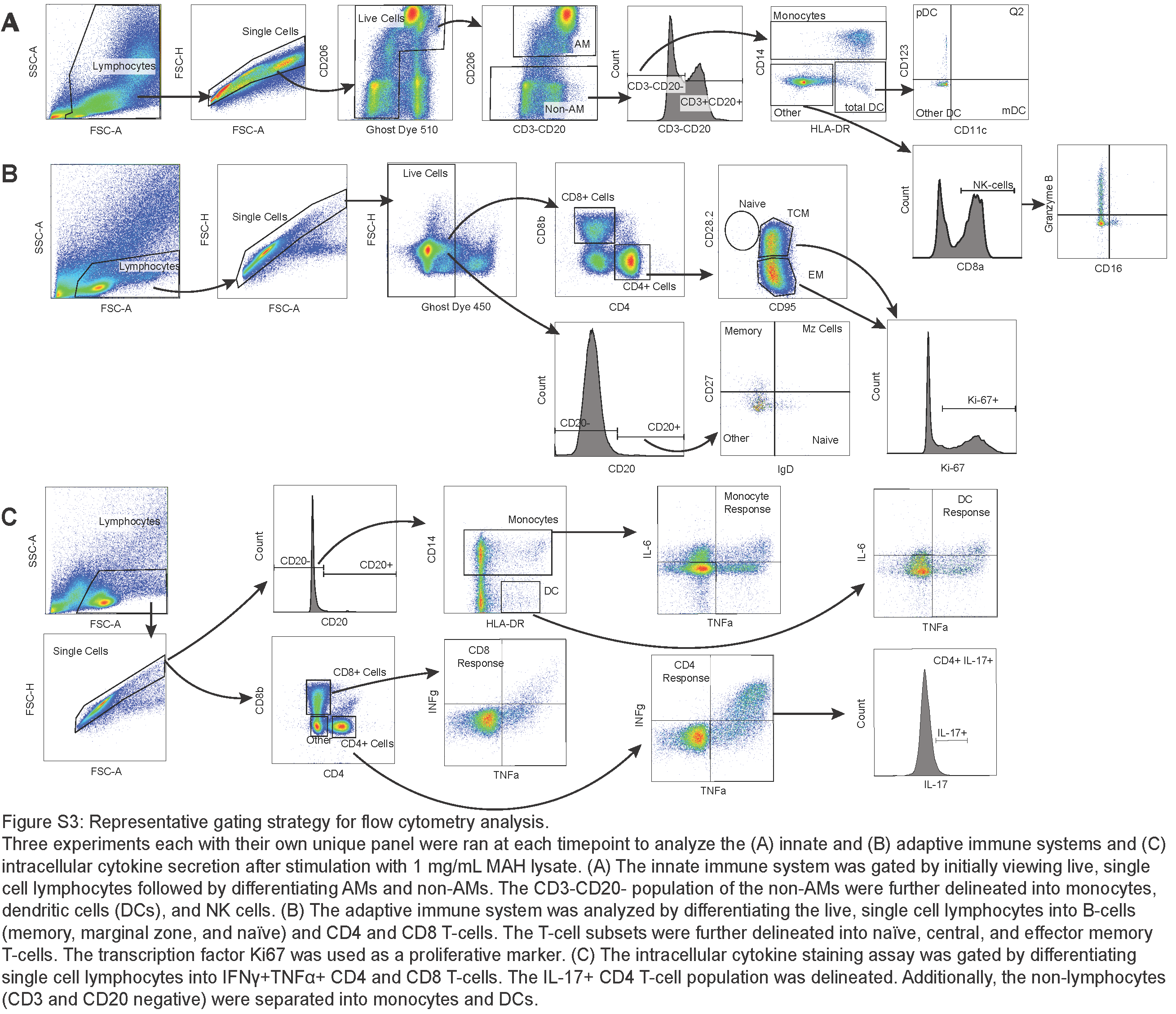

Supplement: Figure S3 — Representative gating strategy for flow cytometry analysis. [file mbio.00829-24-s0003.tiff]

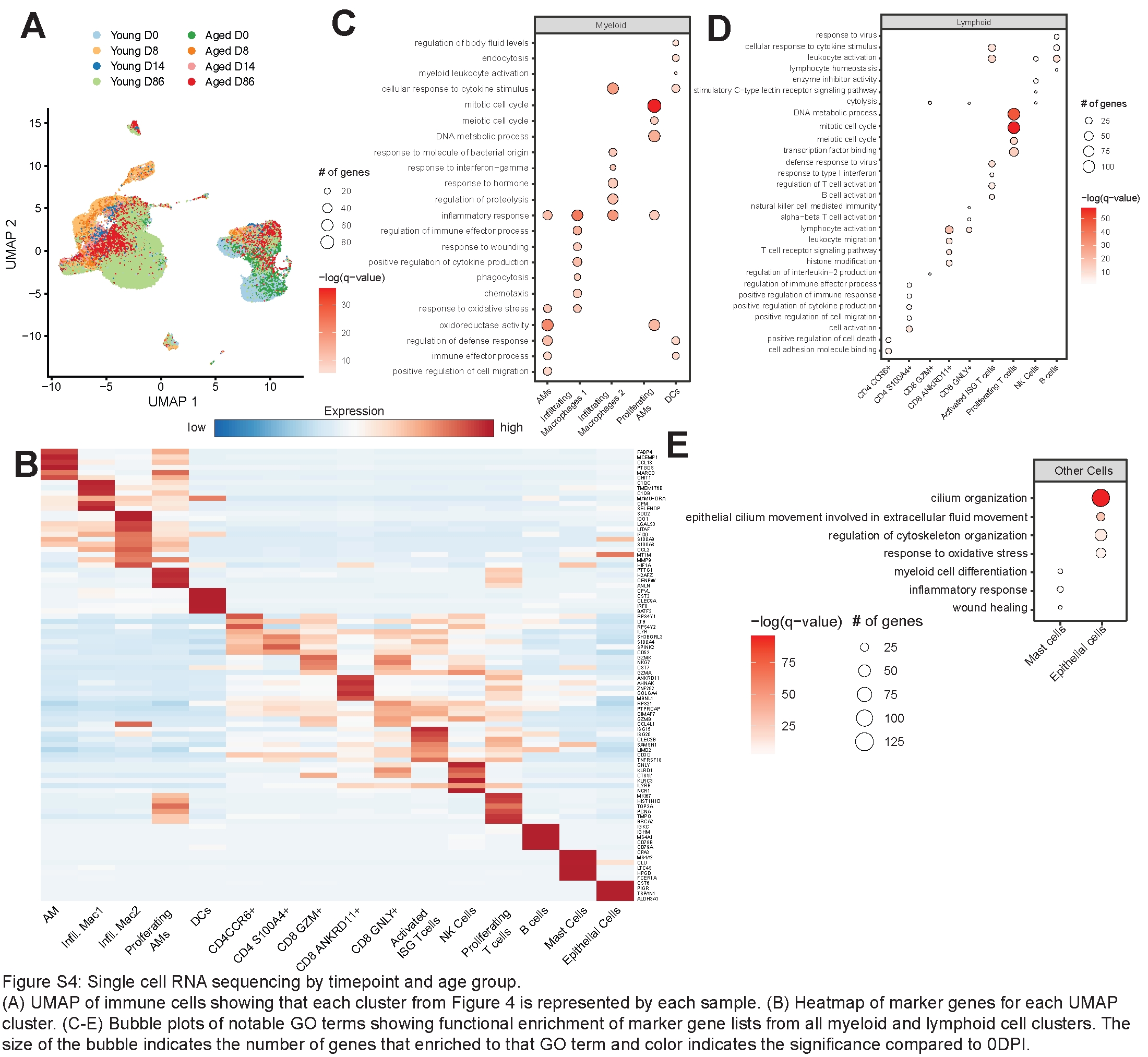

Supplement: Figure S4 — Single-cell RNA sequencing by time point and age group. [file mbio.00829-24-s0004.tiff]

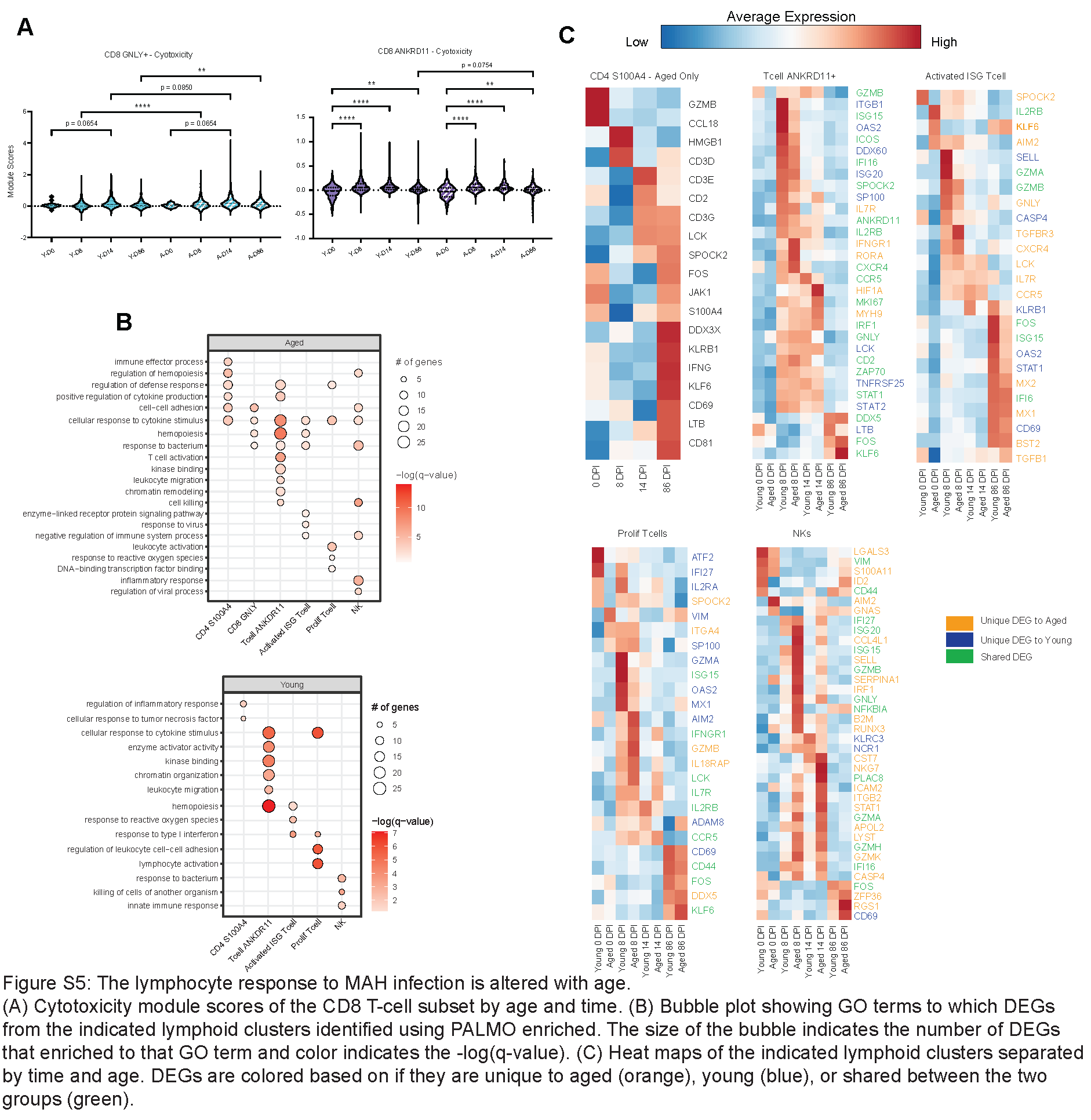

Supplement: Figure S5 — The lymphocyte response to MAH infection is altered with age. [file mbio.00829-24-s0005.tiff]

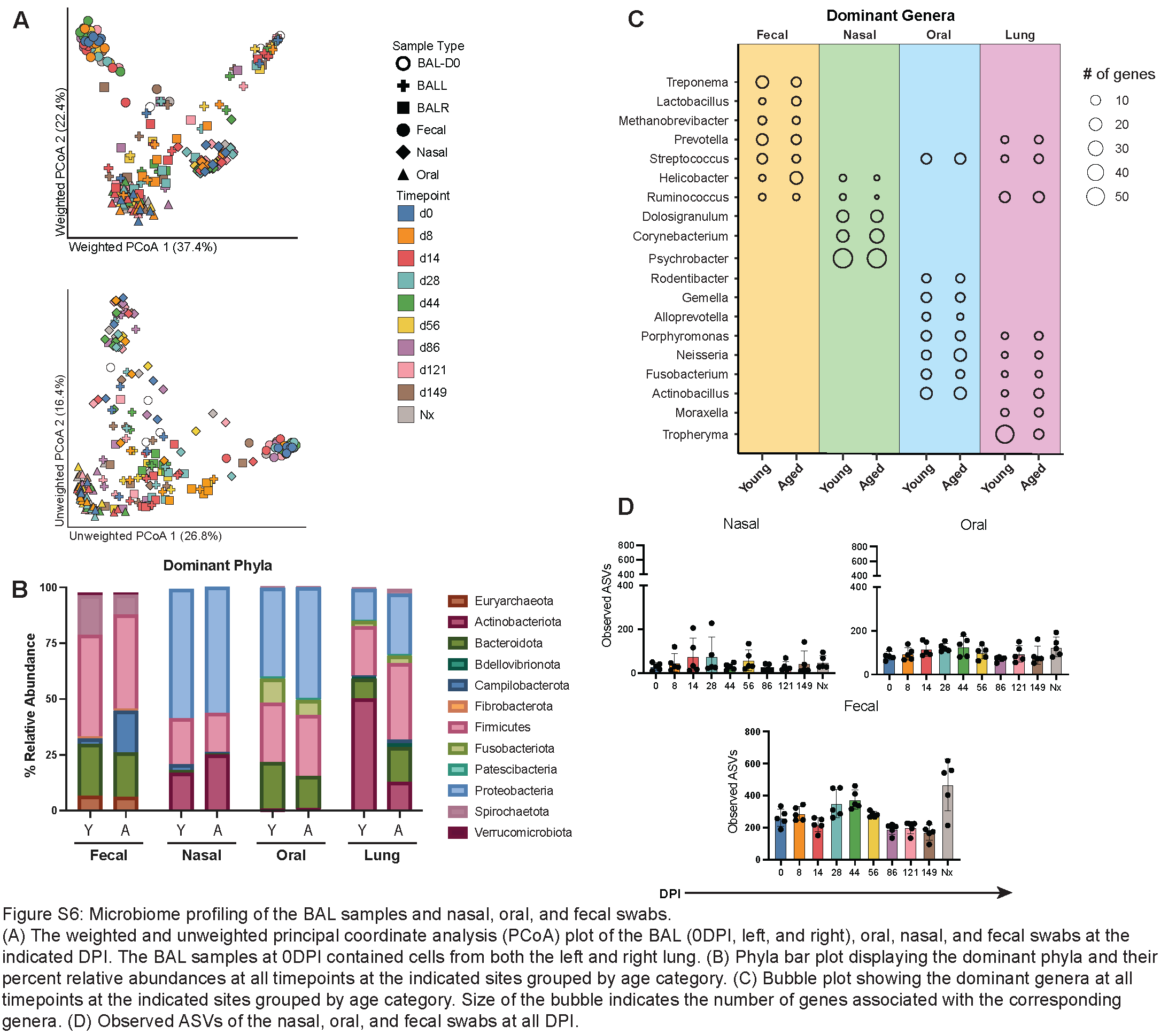

Supplement: Figure S6 — Microbiome profiling of the BAL samples and nasal, oral, and fecal swabs. [file mbio.00829-24-s0006.tiff]
