# Supplementary material for: Immunological and microbial shifts in the aging rhesus macaque lung during nontuberculous mycobacterial infection
Source: mBio. 2024 May 21;15(6):e00829-24. doi: 10.1128/mbio.00829-24 (PMC11237422; doi:10.1128/mbio.00829-24)
Supplement: Table S1 — Metadata of the research animals. [file mbio.00829-24-s0007.docx]

**Table S1: Metadata of the research animals.**

|  | | | | | |
| --- | --- | --- | --- | --- | --- |
| BlindID | Group | Age (y) | M/F | Weight at Inoculation (kg) | Weight at Nx (kg) |
| OR_Adult_3 | Adult | 8.8 | F | 8.70 | 8.45 |
| OR_Adult_8 | Adult | 6.1 | M | 7.10 | 7.45 |
| OR_Adult_10 | Adult | 5.7 | M | 8.00 | 7.10 |
| OR_Aged_12 | Aged | 21.0 | F | 7.80 | 8.05 |
| OR_Aged_32 | Aged | 23.9 | F | 8.65 | 9.10 |
