# Supplementary material for: Immunological and microbial shifts in the aging rhesus macaque lung during nontuberculous mycobacterial infection
Source: mBio. 2024 May 21;15(6):e00829-24. doi: 10.1128/mbio.00829-24 (PMC11237422; doi:10.1128/mbio.00829-24)
Supplement: Table S2 — The weighted intragroup Unifrac distances showing β-diversity at each time point and comparing to baseline (0DPI) for the right and left BAL. [file mbio.00829-24-s0008.docx]

**Table S2: The weighted intragroup Unifrac distances showing β-diversity at each time point and comparing to baseline (0DPI) for the right and left BAL.**

| **Unifrac Distance BAL Right Intragroup (Weighted)** | | | | | | |  |  |  |
| --- | --- | --- | --- | --- | --- | --- | --- | --- | --- |
| **D0** | **D8*** | **D15** | **D28** | **D44** | **D56** | **D86** | **D121** | **D149** | **Nx** |
| 0.0929 | 0.2071 | 0.4808 | 0.4013 | 0.4501 | 0.4022 | 0.2917 | 0.5746 | 0.5640 | 0.0589 |
| 0.4518 | 0.1135 | 0.1467 | 0.1489 | 0.1112 | 0.2353 | 0.3475 | 0.1905 | 0.3799 | 0.4802 |
| 0.5210 | 0.1850 | 0.5226 | 0.4674 | 0.4936 | 0.4636 | 0.4326 | 0.5717 | 0.5162 | 0.4771 |
| 0.0912 | 0.1331 | 0.1734 | 0.1973 | 0.4364 | 0.2934 | 0.3333 | 0.2791 | 0.4095 | 0.1097 |
| 0.0045 | 0.1105 | 0.3667 | 0.2945 | 0.0282 | 0.5566 | 0.4250 | 0.3528 | 0.3942 | 0.0797 |
| 0.5200 | 0.1062 | 0.1987 | 0.2329 | 0.4802 | 0.3277 | 0.2660 | 0.3201 | 0.1510 | 0.5104 |
| 0.5119 | 0.0787 | 0.2519 | 0.2069 | 0.1204 |  |  | 0.1255 | 0.3170 |  |
| 0.5822 | 0.2225 | 0.4091 | 0.4295 | 0.4714 |  |  | 0.5847 | 0.5089 |  |
| 0.3209 | 0.1016 | 0.2666 | 0.2025 | 0.1208 |  |  | 0.1413 | 0.1692 |  |
| 0.5815 | 0.1456 | 0.2679 | 0.1876 | 0.4586 |  |  | 0.2939 | 0.2205 |  |
|  |  |  |  |  |  |  |  |  |  |
| **Unifrac Distance BAL Left Intragroup (Weighted)** | | | | | | |  |  |  |
| **D0** | **D8** | **D15** | **D28** | **D44** | **D56** | **D86** | **D121** | **D149** | **Nx** |
| 0.0915 | 0.2933 | 0.3803 | 0.3849 | 0.1607 | 0.3018 | 0.0008 | 0.5726 | 0.2597 | 0.0568 |
| 0.4538 | 0.1272 | 0.2553 | 0.3868 | 0.3205 | 0.2502 | 0.3172 | 0.1471 | 0.3437 | 0.4825 |
| 0.5220 | 0.2562 | 0.5702 | 0.0060 | 0.4083 | 0.4381 | 0.3174 | 0.4380 | 0.4734 | 0.4772 |
| 0.0900 | 0.5042 | 0.3751 | 0.3449 | 0.2587 | 0.2871 | 0.4504 |  | 0.4183 | 0.1084 |
| 0.0048 | 0.2304 | 0.0303 | 0.4586 | 0.2233 | 0.2312 | 0.4509 |  | 0.1753 | 0.0808 |
| 0.5211 | 0.4712 | 0.5629 | 0.4607 | 0.5534 | 0.4769 | 0.4657 |  | 0.5784 | 0.5101 |
| 0.5122 | 0.1680 | 0.2228 | 0.2371 | 0.2870 | 0.2971 | 0.0026 |  | 0.1501 |  |
| 0.5809 | 0.3699 | 0.4498 | 0.3697 | 0.3545 | 0.4837 | 0.0027 |  | 0.3282 |  |
| 0.3206 | 0.2394 | 0.2804 | 0.3703 | 0.1295 | 0.0642 | 0.3154 |  | 0.2252 |  |
| 0.5805 | 0.5634 | 0.4445 | 0.5401 | 0.5162 | 0.5257 | 0.4488 |  | 0.4852 |  |
|  |  |  |  |  |  |  |  |  |  |
| * = p ≤ 0.05 when compared to D0 | | | | |  |  |  |  |  |
