# Supplementary material for: Immunological and microbial shifts in the aging rhesus macaque lung during nontuberculous mycobacterial infection
Source: mBio. 2024 May 21;15(6):e00829-24. doi: 10.1128/mbio.00829-24 (PMC11237422; doi:10.1128/mbio.00829-24)
Supplement: Table S3 — Collection timeline of the various experimental readouts. [file mbio.00829-24-s0009.docx]

| **Table S3: Collection timeline of the various experimental read-outs** |
| --- |

| Experimental Parameter | Timepoints collected (days post-infection; DPI) | Lungs tested |
| --- | --- | --- |
| Mycobacterial Culturing | 0, 8, 14, 28, 44, 56, 86, 121, 149, 190 | Right and Left |
| quantitative PCR | 0, 8, 14, 28, 44, 56, 86, 121 | Right and Left |
| Computed tomography | 0, 13, 41, 83, 118, 146, 183 | Right and Left |
| Immune mediator production (Luminex) | 0, 8, 14, 28, 89, 121, 145, 184 | Right and Left |
| Flow Cytometry | 0, 8, 14, 28, 44, 56, 86, 121, 149, 190 | Right and Left |
| Single cell RNA sequencing | 0, 8, 14, 86 | Right and Left |
| Microbiome profiling | 0, 8, 14, 28, 44, 56, 86, 121, 149, 190 | Right and Left |
